# Genome-wide association study to identify genomic loci associated with early vigor in bread wheat under simulated water deficit complemented with quantitative trait loci meta-analysis

**DOI:** 10.1093/g3journal/jkac320

**Published:** 2022-12-02

**Authors:** Yousef Rahimi, Bahman Khahani, Ali Jamali, Hadi Alipour, Mohammad Reza Bihamta, Pär K Ingvarsson

**Affiliations:** Department of Plant Biology, Uppsala BioCenter, Linnean Centre for Plant Biology in Uppsala, Swedish University of Agricultural Sciences, 75007 Uppsala, Sweden; Department of Plant Genetics and Production, College of Agriculture, Shiraz University, 71441-65186 Shiraz, Iran; Department of Agronomy and Plant Breeding, Faculty of Agriculture, University of Tehran, 31587-77871 Karaj, Iran; Department of Plant Breeding and Biotechnology, Faculty of Agriculture, Urmia University, 5756151818 Urmia, Iran; Department of Agronomy and Plant Breeding, Faculty of Agriculture, University of Tehran, 31587-77871 Karaj, Iran; Department of Plant Biology, Uppsala BioCenter, Linnean Centre for Plant Biology in Uppsala, Swedish University of Agricultural Sciences, 75007 Uppsala, Sweden

**Keywords:** GWAS, FarmCPU, MQTL, wheat, water deficit, early vigor, plant genetics and genomics

## Abstract

A genome-wide association study (GWAS) was used to identify associated loci with early vigor under simulated water deficit and grain yield under field drought in a diverse collection of Iranian bread wheat landraces. In addition, a meta-quantitative trait loci (MQTL) analysis was used to further expand our approach by retrieving already published quantitative trait loci (QTL) from recombinant inbred lines, double haploids, back-crosses, and F_2_ mapping populations. In the current study, around 16%, 14%, and 16% of SNPs were in significant linkage disequilibrium (LD) in the A, B, and D genomes, respectively, and varied between 5.44% (4A) and 21.85% (6A). Three main subgroups were identified among the landraces with different degrees of admixture, and population structure was further explored through principal component analysis. Our GWAS identified 54 marker-trait associations (MTAs) that were located across the wheat genome but with the highest number found in the B sub-genome. The gene ontology (GO) analysis of MTAs revealed that around 75% were located within or closed to protein-coding genes. In the MQTL analysis, 23 MQTLs, from a total of 215 QTLs, were identified and successfully projected onto the reference map. MQT-YLD4, MQT-YLD9, MQT-YLD13, MQT-YLD17, MQT-YLD18, MQT-YLD19, and MQTL-RL1 contributed to the highest number of projected QTLs and were therefore regarded as the most reliable and stable QTLs under water deficit conditions. These MQTLs greatly facilitate the identification of putative candidate genes underlying at each MQTL interval due to the reduced confidence of intervals associated with MQTLs. These findings provide important information on the genetic basis of early vigor traits and grain yield under water deficit conditions and set the foundation for future investigations into adaptation to water deficit in bread wheat.

## Introduction

Bread wheat (*Triticum aestivum* L.) is one of the most important staple crops in the world and plays an important role for human consumption (Curtis and Halford, 2014; Appels *et al*., 2018). Food security has been severely impacted by global climate change through increasing average temperatures and accompanying droughts (Curtis and Halford, 2014). Great efforts have therefore been made in most major crops, and particularly in wheat, to address concerns stemming from climate change (Raza *et al*., 2019). Climate change is expected to increase water deficit conditions in many parts of the world and wheat breeding programs have hence put a lot of emphasis on developing new varieties that are better adapted to water limiting conditions without any significant losses in grain yield and biomass (Richards *et al*., 2014; Raza *et al*., 2019). Furthermore, water deficits can impose serious negative consequences on wheat at several different developmental stages, ranging from germination to grain filling (Richards *et al*., 2014; Raza *et al*., 2019).

In many parts of the world, particularly in Mediterranean climate regions, wheat production is limited by inadequate precipitation at lateral growth stages (anthesis to grain filling), usually referred to as terminal drought (Richards, 1991; Rebetzke *et al*., 2007; Savin *et al*., 2015; Zhao *et al*., 2019a). However, higher rainfalls are recorded during autumn and winter which overlap with the early growth of winter wheat in these areas (Rebetzke *et al*., 2007). Previous research has shown that greater early vigor (faster early leaf area development) results in faster seedling establishment and a more rapid canopy closure which reduce soil evaporation and increase nutrient uptake in drought-prone regions, and this ultimately causes higher biomass and grain production (Liao *et al*., 2004; Bertholdsson, 2005; Ludwig and Asseng, 2010; Ryan *et al*., 2015; Vukasovic *et al*., 2022). On the other hand, rapid early growth may also lead to a reduction in available water reservoirs prior to critical developmental stages such as anthesis, heading, and grain filling which has then detrimental effects on grain yield (Richards and Townley-Smith, 1987; Zhao *et al*., 2019a). This happens specifically when greater vegetative growth results in an increased number of nonfertile tillers and a wider transpiration area in leaves (Asseng and Van Herwaarden, 2003). The advantages of rapid early vigor still prevail over the possible disadvantages since highly vigorous wheat genotypes have a higher potential of absorbing water from deeper soil layers due to deeper roots, greater tolerance to water deficits and a quicker recovery in case of erratic water availability (Botwright *et al*., 2002; Asseng and Van Herwaarden, 2003). However, we still lack of information about genomic loci underlying early viogurm which limit the implementation of this trait in breeding programs (Rebetzke *et al*., 2017).

Genome-wide association studies (GWASs) has emerged as promising method to dissect the genetic architecture of quantitative traits (Rahimi *et al*., 2019; Bilgrami *et al*., 2020). The GWAS approach has several benefits compared to standard quantitative trait loci (QTL)-based genetic mapping, including an increased extent of genetic diversity in the mapping population, more cost-effective methods for genotyping and the possibility to transfer results to other landraces, elite cultivars, and advanced breeding lines (Neumann *et al*., 2011; Chen *et al*., 2017b; Rahimi *et al*., 2019; Bilgrami *et al*., 2020). GWAS is thus a complementary approach to QTL mapping for detecting putative candidate genes (CGs) and alleles based on existing patterns of linkage disequilibrium (LD) in a species (Zhu *et al*., 2018; Rahimi *et al*., 2019; Bilgrami *et al*., 2020). In GWAS, the higher mapping resolution stems from taking advantage of naturally occurring recombination within a germplasm collection (Daware *et al*., 2017). GWAS studies have been performed in wheat on traits such as seedling emergence and tillering (Chen *et al*., 2017b), root attributes (Beyer *et al*., 2019), coleoptile length (Sidhu *et al*., 2020), and other relevant agronomic traits (Rahimi *et al*., 2019), and many QTLs have been identified that can be exploited in marker-assisted selection (MAS) breeding programs. However, most of the associations identified to date remain to be functionally validated or even validated in other mapping populations and environments.

On the other hand, a meta-analysis of previously identified QTLs, MQTL analysis, can be useful for identifying genomic regions that are consistently involved in controlling the traits under investigations and for narrowing the confidence intervals (CIs) of the QTL locations (Quraishi *et al*., 2011; Chen *et al*., 2017a; Khahani *et al*., 2019). Integrating results from MQTL and GWAS analyses have also been implemented in a several species, including maize (Chen *et al*., 2017a; Zhu *et al*., 2018), rice (Daware *et al*., 2017; Yang *et al*., 2020a) and wheat (Bilgrami *et al*., 2020). Combining MTQL and GWAS results have been highly effective for identifying CGs and potential genomic regions causally involved in controlling the traits under investigation (Chen *et al*., 2017a; Daware *et al*., 2017; Yang *et al*., 2020b; Bilgrami *et al*., 2020).

In this study, a genome-wide analysis was performed using FarmCPU method on primary growth-related traits including germination attributes, and grain yield under water-deficit conditions (YLD) among Iranian wheat landraces. These results will lay the foundation for future identification of genetic mechanisms alleviating the adverse effects of water deficit on yield and biomass in wheat. Furthermore, a MQTL study was conducted to complement the GWAS and increase the number of genomic regions that are reliably associated with the traits of interest. These approaches help unravel hotspot genomic regions that are consistently associated with investigated traits under unfavorable conditions.

## Material and methods

### Plant material and experimental conditions

From a previously studied collection of bread wheat (Rahimi *et al*., 2019), 100 landraces were selected based on their drought tolerance indices and were evaluated together with four check varieties (Supplementary Table S1) under simulated water deficit conditions by the application of polyethylene glycol 6000 (PEG 6000). First, in a pilot experiment performed to determine the proper concentration of PEG 6000, a subsample of 20 accessions were studied by germinating 30 seeds from each accession in three Petri dishes together with 10 ml of either distilled water (control) or various concentrations of PEG 6000. The concentrations of PEG 6000 corresponded to osmotic potentials of −4, −6, −8, −10, and −12 bars that were calculated using the following formula (Michel and Kaufmann 1973).


$$\mathrm{WP}=-(1.18e-2)C-(1.18e-4)C^{2}+(2.67e-4)CT+(8.39e-7)C^{2}T$$

where WP is osmotic potential of a PEG-6000 solutions (bars), C is the concentration of PEG-6000 in g/kg H_2_O, and T is the temperature in degrees (20°C in the current study).

Seeds were germinated in a germination incubator at 20°C and germinated seeds were counted daily for 10 days until no new germination was recorded in the three replicates of each treatment for 3 consecutive days. The germination criterion we used was when the radicle had protruded at least to a length of ≥2 mm (ISTA, 1999). Primary growth-related traits recorded in seedlings including total fresh weight (TFW), total dry weight (TDW), shoot length (SL), root length (RL), germination rate/speed (GR), total germination percentage (TGP), and normal germination percentage (NGP). The results of the pilot study showed that −10 bars resulted in the greatest variation among accessions while at the −12 bars some of the accessions did not germinate at all (Supplementary Table S2). The remaining 80 accessions used in the study were therefore only evaluated under −10 bars and control conditions with the same germination criterion as described above. The phenotypic dataset used in the GWAS is supplied as Supplementary Table S3.

### SNP genotyping, population structure, and individual relationship

As previously described by Alipour *et al*. (2017), a genotyping-by-sequencing (GBS) protocol was used to generate sequencing data from genomic DNA. Briefly, after DNA extraction and quality control, GBS libraries were constructed according to the protocol of Poland *et al*. (2012). Two restriction enzymes *PstI* and *MspI* were used to digest genomic DNA, and T4 ligase was used to ligate adaptors. The concentration of libraries was then estimated on a Qubit 2.0 fluorometer after the selection of fragments in the range of 250–330 bp using an E-gel system. Libraries were sequenced in an Ion Proton sequencer. Sequencing reads were trimmed to 64 bp and grouped into sequence tags and SNPs were called using internal alignment by allowing mismatches of up to 3 bp. The SNP calling was carried out through the GBS pipeline Universal Network Enabled Analysis Kit, by filtering reads with low-quality score (<15) and low minor allele frequency <1% to reduce false-positive markers. The relationship between landraces was determined using the VanRaden Method implemented in rMVP or Memory-efficient, Visualization-enhanced, and Parallel-accelerated Tool (Yin *et al*., 2021) in RStudio. Population structure was analyzed by calculating principal components (PCs) based on 10,938 SNP markers. To estimate variance components, the default method (Brent) was used in rMVP.

### GWAS

Fixed and random model with Circulating Probability Unification (FarmCPU) was employed to perform GWAS using the rMVP package in R (Yin *et al*. 2021). The FarmCPU model provides greater statistical power compared to GLM and MLM and alleviates the problem of confounding effects and false positives at the same time. This occurs by fitting iteratively detected associated markers as cofactors to test the rest markers in a fixed effect model while a random effect model is used to select the associated markers based on a maximum likelihood method to prevent model overfitting. Manhattan plots were used to visualize associations between phenotype and genotype from the GWASs. In these plots, SNPs are ordered based on their chromosome and base-pair positions along the x-axis while the y-axis display the negative logarithm of the *P*-value generated from the GWAS F-test for testing H_0_, i.e. no association between marker genotypes and the phenotype.

### Gene descriptions and pathway analysis

Genomic regions surrounding all significantly associated SNPs were retrieved and the Gramene database was used to assign gene annotations by aligning the genomic regions to the IWGSC RefSeq v1.0 annotation (https://wheat-urgi.versailles.inra.fr/Seq-Repository/Annotations). The functions of the CGs were assessed using the pathway's descriptions. Overlapping genes with the highest identity percentage and blast score were selected for further processing. The gene ontology (GO) descriptions were obtained from EnsemblPlants database (http://plants.ensembl.org/index.html). The sequences from homologous genes in rice genes were obtained using Ensembl Plants BioMart. The KOBAS software (Xie *et al*., 2011) was then used to determine enriched pathways using the Kyoto Encyclopedia of Genes and Genomes (KEGG) database.

### Phenotypic data analysis

Phenotypic data were analyzed using SAS v.9.4 and adjusted means were estimated based on the alpha-lattice design using GLM and Mixed procedures and were used for advanced linear analysis of YLD and drought tolerance indices (Supplementary Table S4).

### QTL studies used for projections and MQTL analysis

To improve and enrich our results for identifying putative CGs, an inclusive literature survey was conducted on wheat QTLs studies related to YLD and other traits under water deficit conditions published from 2007 to 2021 (Supplementary Table S5). Thus, QTLs for YLD, germination attributes, TDW, RL, SL, and TFW under water deficit conditions were collated on the different chromosomes of wheat from 52 bi-parental wheat populations extracted from 49 studies. QTL studies without proper genetic map information or QTL-related information were discarded from this study. The wheat reference map from Liu *et al*., (2020b) is the most comprehensive and saturated genetic map currently available and was selected for our analysis. QTLs with sufficient data, including QTL position, chromosome groups, CI, the proportion of phenotypic variance (R^2^), and log of odds ratio (LOD) were collected from the 52 populations with BioMercator v4.2 and used in the MQTL analysis. To estimate 95% CI for QTLs, the formulas reported by Darvasi *et al.*, (1997) and Guo *et al*. (2006) was used.

After the projection of QTLs from different populations onto the reference map, the MQTL analysis was implemented on the integrated and re-positioned QTLs using BioMercator V4.2 (Arcade *et al*., 2004; Veyri*et al.*, 2007; Sosnowski *et al*., 2012). The most likely model of MQTLs was selected among different models in BioMercator based on the Akaike Information Criterion (AIC), the corrected AIC (AICc and AIC3), the Bayesian Information Criterion, and the Average Weight of Evidence criteria. The position of MQTLs and related QTLs and the MQTL and QTL distributions on the reference map were displayed using *SOFIA* (Diaz-Garcia *et al*., 2017). In addition to the genetic position of MQTLs, the distribution of MQTLs across the wheat genome (IWGSC) along chromosomes was investigated and displayed using heatmaps with the *Pheatmap* and *RIdeogram* packages (Kolde, 2013; Hao *et al*., 2020). Additionally, to expand our genomic approaches, gene density and SNP variation across the wheat chromosomes were obtained from the EnsemblPlants database (http://plants.ensembl.org/index.html). The distribution of all these variables discussed above, including the MQTL genomic positions, gene density, and SNP density, were also visualized using Circos (Krzywinski *et al*., 2009).

To identify genes underlying for the studied traits that located in the corresponding regions of the identified MQTLs, the genomic position of flanking markers for each MQTL was retrieved from T3/Wheat database (https://triticeaetoolbox.org/wheat/). The gene annotations from all MQTL genomic regions were carefully explored in the EnsemblPlants database (http://plants.ensembl.org/index.html). Furthermore, the orthologous genes situated at each MQTL interval were surveyed in rice and functional CGs were identified according to their function in rice. The accessible annotation of genes in rice (https://funricegenes.github.io/) was used for identifying putative CGs based to orthologous genes of wheat in rice (Huang *et al*., 2022). Four studies (Derakhshani *et al*., 2020; Rahimi *et al*., 2021; Konstantinov *et al*., 2021; Nouraei *et al*., 2022) related to the transcriptomics of wheat against water deficit conditions were used to compare the differential expressed genes with the genes retrieved from MQTL and GWAS analyses. The KOBAS software (Xie *et al*., 2011) was used to detect enriched pathways for the common genes between these three different databases.

## Results

### Phenotypic diversity in seedling traits

Descriptive statistics from both field trials and simulated water deficit experiments are summarized in Table 1, where mean, range, broad-sense heritabilities, and correlation between traits are displayed. Based on field trial results, the average grain yield across all landraces decreased from 1.90 g per spike under normal irrigation (N-YLD) to 0.97 g per spike under drought conditions (YLD). The maximum variance was observed for NGP (Std: 10.14), and the minimum was observed for the TDW (SD 0.01). The highest heritability was estimated for GR (80.38), while YLD showed the lowest heritability (62.86). Correlation analysis showed that there was a significant positive association between NGP and TDW and GR (*r^2^* = 0.24**, *r^2^* = 0.29**, respectively). However, the highest correlation among early vigor traits was observed between TGP and NGP (*r^2^* = 0.91**), GR and TDW (*r^2^* = 0.60**), GR and SL and RL (*r^2^* = 0.53**, *r^2^* = 0.46**). Interestingly, all characterized early vigor traits had positive but non-significant correlations with YLD, while they had positive and significant correlations with N-YLD. Furthermore, drought tolerance indices were also positively correlated with GR, TFW, TDW, SL and RL (Table 1).

**Table 1.** Summary statistics for germination parameters, heritabilities and correlations among traits for bread wheat landraces evaluated under PEG-induced water deficit conditions.

Trait	Mean	Range	SD	H^2^	TGP (%)	NGP (%)	GR (gs per time*^a^*)	TFW (g per seedling)	TDW (g per seedling)	SL (cm)	RL (cm)	YLD (g per spike)	N-YLD (g per spike)	GMP	STI
TGP (%)	94.90	35.00	6.65	77.14	1.00	0.91*^b^*	0.32*^b^*	0.18	0.33*^b^*	−0.07	0.07	0.09	0.08	0.09	0.08
NGP (%)	92.79	70.00	10.14	79.32		1.00	0.29*^b^*	0.15	0.24*^b^*	−0.16	0.04	0.07	0.11	0.10	0.08
GR (gs per time*^a^*)	0.44	0.71	0.10	80.38			1.00	0.59*^b^*	0.60*^b^*	0.53*^b^*	0.46*^b^*	0.17	0.22*^c^*	0.22*^c^*	0.23*^c^*
TFW (g per seedling)	0.23	0.47	0.07	78.66				1.00	0.58*^b^*	0.28*^b^*	0.52*^b^*	0.06	0.41*^b^*	0.23*^c^*	0.22*^c^*
TDW (g per seedling)	0.04	0.05	0.01	85.4					1.00	0.52*^b^*	0.53*^b^*	0.10	0.30*^b^*	0.21*^c^*	0.23*^c^*
SL (cm)	1.55	4.74	1.08	78.59						1.00	0.48*^b^*	0.17	0.22*^c^*	0.22*^c^*	0.26*^b^*
RL (cm)	7.22	10.41	2.05	72.34							1.00	0.15	0.30*^b^*	0.25*^b^*	0.27*^b^*
YLD (g per spike)	0.97	1.42	0.31	62.86								1.00	0.43*^b^*	0.88*^b^*	0.88*^b^*
N-YLD (g per spike)	1.90	2.55	0.46	66.66									1.00	0.79*^b^*	0.79*^b^*
GMP	1.34	1.35	0.32	-										1.00	0.99*^b^*
STI	0.49	1.03	0.23	-											1.00

gs per rime: germinated seeds in the time interval.

Significant at *P* < 0.001.

Significant at *P* < 0.05.

H^2^, heritability; GRTFW, total fresh weight; N-YLD, grain yield under normal irrigation in the field; GMP, geometric mean productivity which is calculated based on field data; STI, stress tolerance index which is calculated based on field data.

### Genetic markers and population structure

**Fig. 1.**
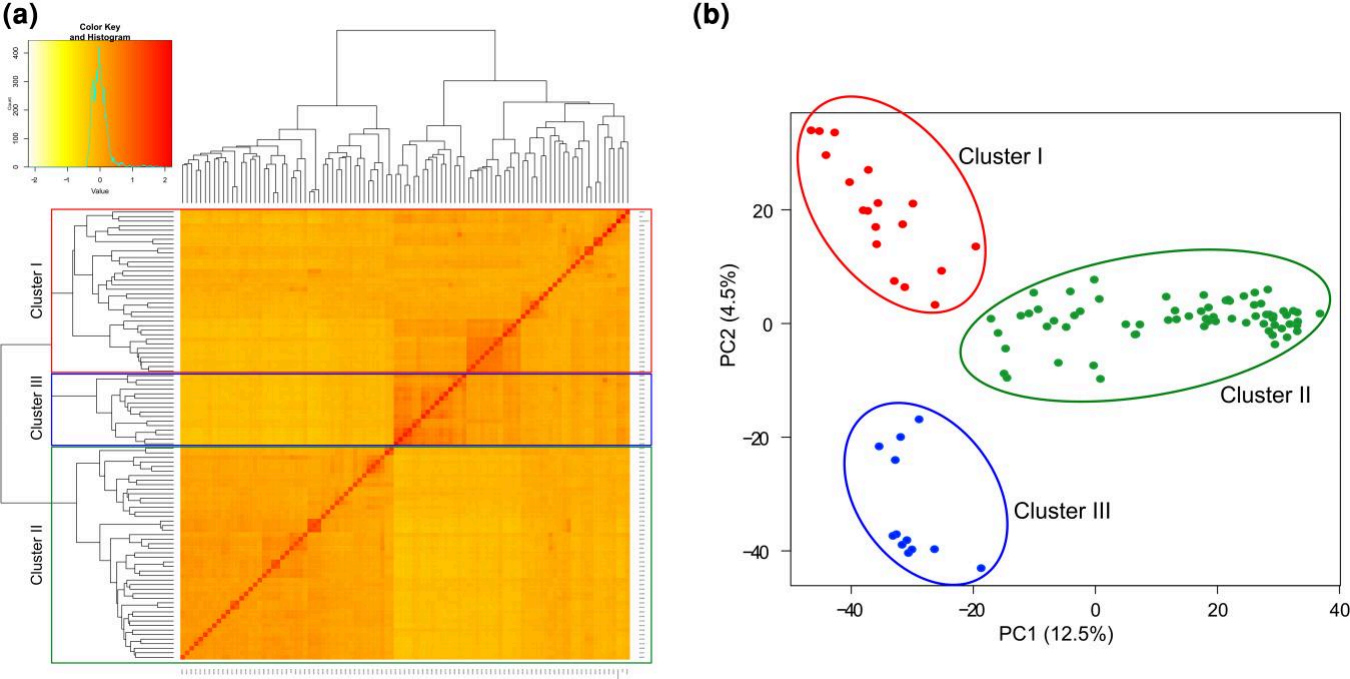
Population structure of Iranian wheat landraces. a) cluster analysis using Kinship matrix, and b) PCA using PC1 and PC2. Cluster I constituted of landraces originating mainly from the Northern, central, and western areas in Iran together with three cultivars Kaveh, Naz, and Koohdasht. Cluster II represents the biggest group of landraces from different regions across Iran. Cluster III represents the smallest group of landraces originating mainly from the central and western areas in Iran.

Most DNA substitutions were transitions (A↔G and T↔C), and the transversion rates were significantly lower (Supplementary Table S6). The average Ts/Tv ratio for the A, B, and D genomes were 1.88, 1.76, and 1.40, respectively. The density of markers (SNP/Mbp) across the different genomes also varied with the B genome having the highest average SNP density (0.89), while the D genome had the lowest SNP density (0.41). The highest number of SNPs in three sub-genomes were observed on chromosomes 7A (732), 2B (835), and 2D (385). The average minor allele frequency (MAF) of SNPs, and gene densities were similar in A and B genomes and greater than in the D genome. Furthermore, the A and B genomes showed higher heterozygosities than the D genome, with the overall heterozygosity across the entire whole genome was 0.052. The average polymorphism information content (PIC) was 0.22, 0.23, and 0.21 for A, B, and D genomes, respectively and ranged from 0.16 in 4B chromosome to 0.25 in 6A chromosome. Moreover, the total number of SNP pairs (TNSP) in the whole genome was 493,675, where 47% (230,300) of the TNSPs were located in the B genome alone (Supplementary Table S7). Among SNPs, 16%, 14%, and 16% were in significant LD in the A, B, and D genomes, respectively, and varied between 5.44% (4A) and 21.85% (6A). Three main subgroups among landraces having different degrees of admixture were identified through calculating variance-covariance matrix of individuals (Fig. 1a). The principal component analysis (PCA) found the two first PCs axes explain approximately 13 and 4.5% of the total variation, respectively (Fig. 1b). The PCA confirmed the existence of three subgroups, and these are highlighted in different colors in Fig. 1b.

### Marker-trait associations of early vigor in wheat landraces

**Fig. 2.**
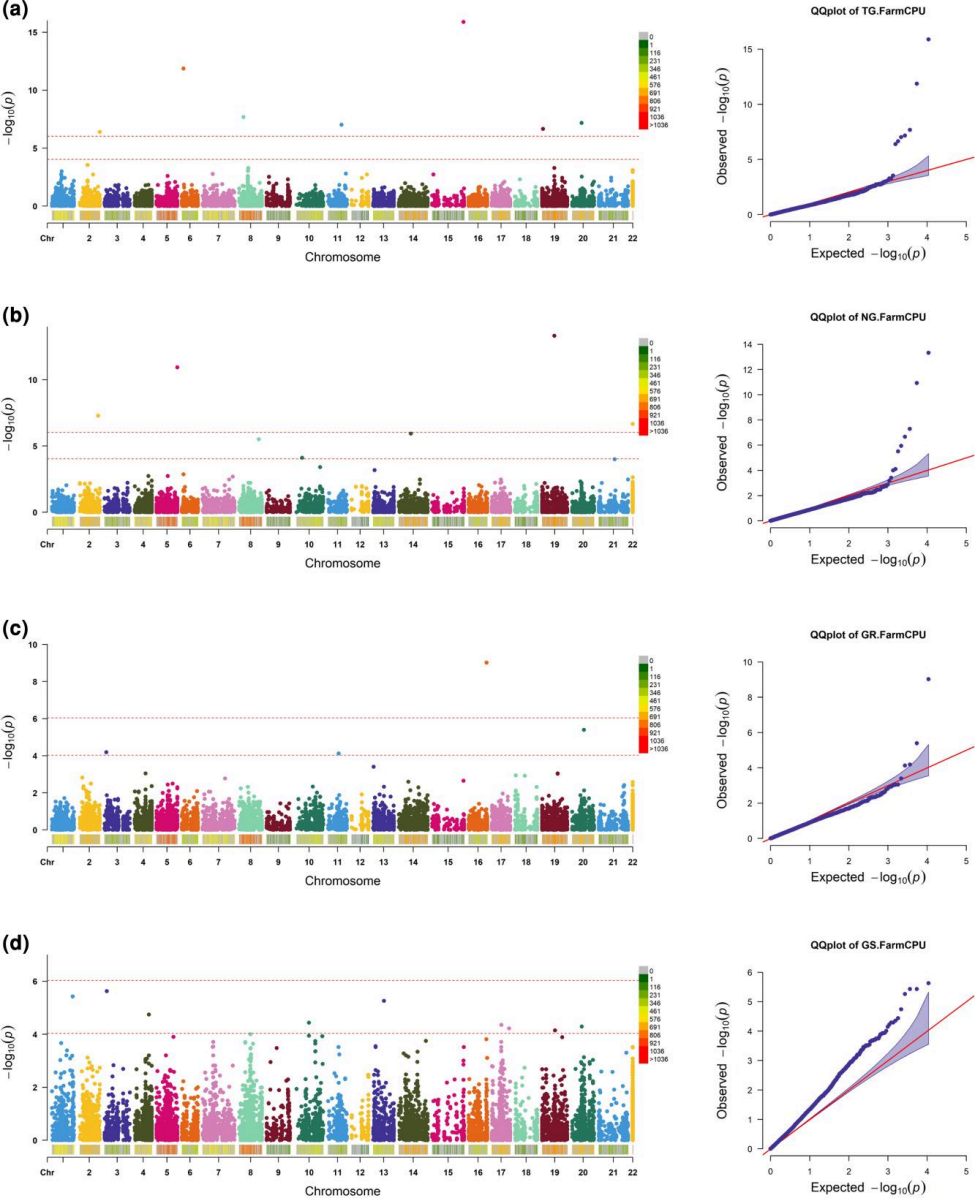
Manhattan and QQ-plots of highly associated SNPs for agronomic traits under water deficit condition. a) TGP, b) NGP, c) germination speed, and d) grain yield under drought stress.

**Fig. 3.**
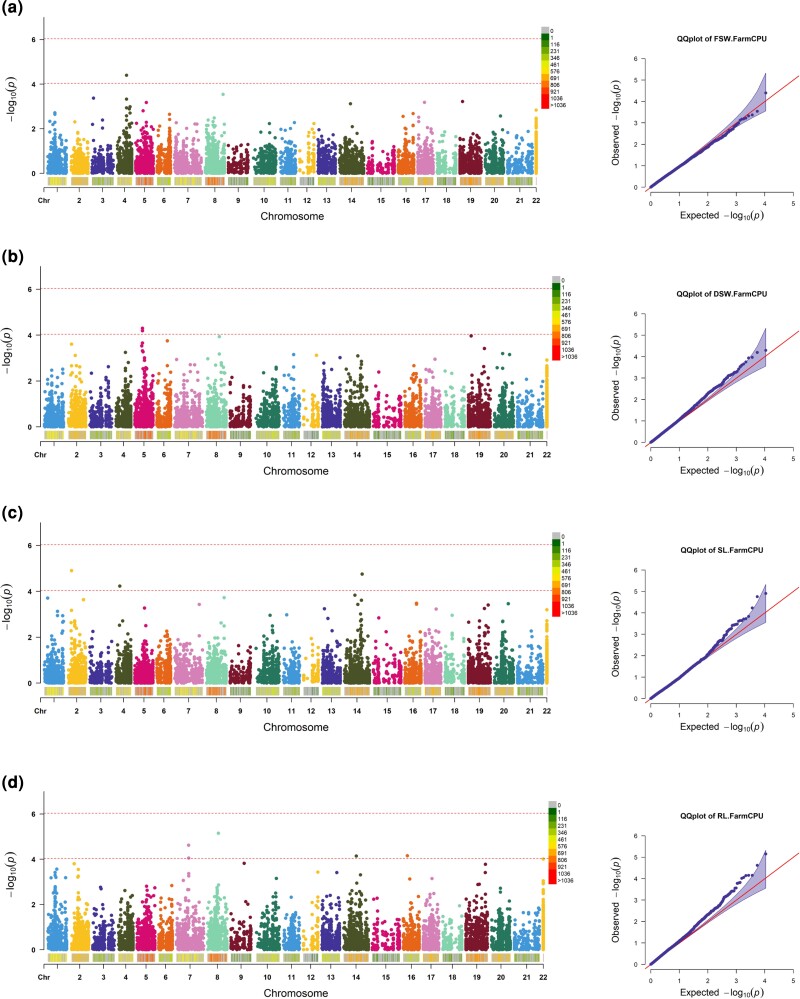
Manhattan and QQ-plots of highly associated SNPs for agronomic traits under water deficit condition. a) seedling fresh biomass, b) seedling dry biomass, c) seedling SL, and d) seedling RL.

For the water deficit condition, 54 highly significant marker-trait associations (MTAs) were identified across all chromosomes with the FarmCPU method at a significance level of –log_10_  *P-*value >4 (Table 2). It is evident that the highest number of MTAs were located in the A genome with 24 MTAs followed by genomes B and D with 23 and 7 MTAs, respectively (Table 2). Moreover, Manhattan and QQ-plots are displayed for all measured traits to help with the identification of markers significantly associated with germination traits and grain yield under simulated water deficit (Figs. 2 and 3). Seven markers, including rs46500, rs10021, rs3901, rs5359, rs44371, and rs33214, were detected for TGP, and seven markers, including rs58619, rs52517, rs2003, rs39056, rs29630, rs20378, and rs5221, were identified for NGP. It is noteworthy to mention that for the normal and TGPs, the B genome contributed to the highest number of MTAs compared to the A and D genomes. TDW accounted for the lowest number of MTAs with a single marker located in the A genome. To evaluate the accuracy of GWAS results under water deficit conditions, QQ-plots were produced to assess that inclusion of population structure and kinship relationships adequately controlled for spurious associations due to population subdivision. The QQ-plots indicated that the observed values largely matched with the expected values, suggesting that our GWAS analyses have adequately controlled for spurious effects due to population structure.

**Table 2.** Marker-trait associations for seed germination traits of bread wheat identified from the different sub-genomes of wheat.

Genome	Early vigor traits in wheat seedlings and stressed-grain yield								
	RL	SL	TFW	TDW	TGP	NGP	GR	YLD	Total
Genome A	4	1	3	1	1	2	1	11	24
Genome B	3	3	1	nf	4	4	2	6	23
Genome D	1	nf	1	nf	2	1	1	1	7
MTA	8	4	5	1	7	7	4	18	54

RL, root length; SL, shoot length; TFW, total fresh weight; TDW, total dry weight; TGP, total germination percentage; NGP, normal germination percentage; GR, germination rate; YLD, grain yield under drought condition; nf, not found.

### Gene annotation and pathway analysis

The GO analyses of the identified MTAs revealed that around 75% were located within or closed to protein-coding genes (Supplementary Table S8). Genes associated with germination traits were mainly involved in biological processes such as cation transport, transmembrane transport, oxidation-reduction process, protein phosphorylation, carbohydrate metabolic process, actin filament organization, organic substance metabolic process, carbohydrate metabolic process, glycogen metabolic process, amylopectin biosynthetic process, starch biosynthetic process, positive regulation of seed germination, and histone H4-R3 methylation. Genes associated with seedling biomass and shoot and RL encode proteins that are involved in intracellular protein transport, regulation of transcription, DNA-templated, snRNA processing, protein phosphorylation, and exocytosis. A chromosomal survey indicated that the A and B genomes harbored the highest number of MTAs (21 and 14, respectively), while only 6 MTAs were located within coding sequences in the D genome. GO enrichment analysis was also performed to identify pathways that were associated with the MTAs and identified significant enrichments for starch and sucrose metabolism and plant hormone signal transduction (Supplementary Table S9). For starch and sucrose metabolism, isoamylase 2 (K01214) and for plant hormone signal transduction, auxin-responsive protein SAUR71-like (K14488), responded to water deficit conditions.

### Characteristics of QTLs in wheat under water deficit condition

To expand our approach for identifying genomic regions controlling our traits of interest, in addition to the GWAS approach, a MQTL analysis was conducted using data on QTLs detected under water deficit (Supplementary Table S7). A total of 215 QTLs controlling YLD, RL, GR, TDW, and TFW in wheat under water deficit condition were retrieved from 52 mapping populations reported in 49 studies published since 2007. The identified QTLs were derived from many different types mapping population types, including 34 recombinant inbred lines (RILs), 14 double haploids (DH), 3 back-crosses (BC), and 1 F_2_ mapping population. The number and distribution of QTLs for each trait across the 21 wheat chromosomes are shown in Supplementary Fig. S1. The QTLs are unevenly distributed across the genomes with the B sub-genome having the highest number of QTLs with 101, followed by A and D sub-genomes with 65 and 49 QTLs, respectively. Chromosome 5B had the highest number of QTLs with 26 QTLs followed by chromosome 2D with 19 and 1B with 18. Chromosome 5D accounted for the lowest number of QTLs with 2 QTLs. Furthermore, YLD and RL harbored the highest number of reported QTLs with 167 and 26 QTLs, respectively. GR, TDW, and TFW had the lowest number of QTLs. The QTLs for YLD were mainly situated on chromosomes 5B, 7A and 1B with 17, 17 and 16 QTLs, respectively. Similarly, in this study, the highest number of QTLs for RL were identified on chromosome 5B.

### Detected MQTLs

**Fig. 4.**
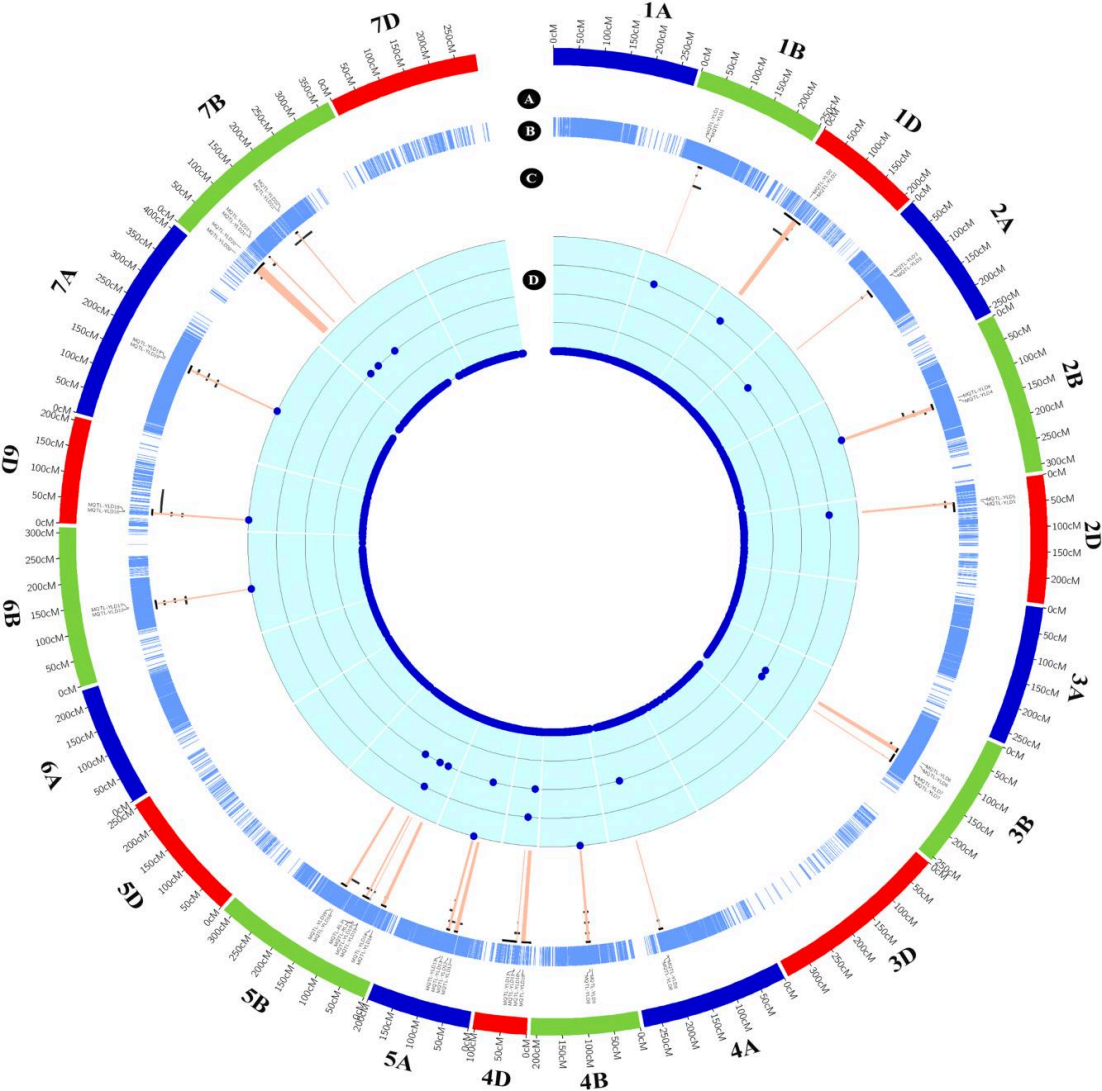
The distribution of a) of MQTLs on the wheat reference map based on the genetic position, b) marker density, c) position of QTLs and MQTL showing through red heatmap across the QTLs, and d) the number of QTLs for each MQTL.

Twenty-three MQTLs were identified from the 215 QTLs that could successfully project onto the reference map (Table 3; Fig. 4). The MQTL analysis substantially restricted the number of QTLs to roughly 10% of identified QTLs, as only MQTLs containing at least 2 QTLs from different studies were considered to improve the accuracy of our analysis. MQT-YLD4, MQT-YLD9, MQT-YLD13, MQT-YLD17, MQT-YLD18, and MQT-YLD19 contributed to the highest number of projected QTLs with four QTLs (Table 3). Intriguingly, these MQTLs might be robust, stable, and suitable candidates for identifying promising QTLs from different locations and years under water deficit conditions. Only one MQTL on chromosome 5B was detected for RL based on our approach. Most of the MQTLs related to the YLD were situated on chromosomes 5B and 7B. The distribution pattern of MQTLs across chromosomes is highly uneven with no MQTLs detected on chromosomes 1A, 3A, 3D, 5D, 6A, and 7D (Table 3; Fig. 4).

**Table 3.** Characteristics of identified MQTLs for grain yield and root length in wheat under water deficit condition.

Trait	Chr.	MQTL	Flanking markers	Position on the consensus reference map (cM)	Confidence interval (cM)	Genomic position on the rice genome (Mb)	Number of initial QTLs	Number of studies	Number of populations	Number of genes underlying the MQTL interval
YLD	1B	MQTL-YLD1	IWA4389-IWB7205	63.24	0.45	326.77–334.54	3	3	3	74
	1D	MQTL-YLD2	wPt-3855-wPt-7035	61.46	8.98	3.34–8.60	3	2	2	136
	2A	MQTL-YLD3	IWB38930-IWB32212	83.55	1.16	348.24–378.84	2	2	2	59
	2B	MQTL-YLD4	IWA3237-IWA2294	128.61	6.74	532.09–591.91	4	3	3	395
	2D	MQTL-YLD5	IWB10124-IWB8481	37.42	2.16	14.86–15.96	3	2	2	30
	3B	MQTL-YLD6	IWB32656-IWB7374	110.14	7.76	760.18–779.36	2	2	2	220
	3B	MQTL-YLD7	IWA3159-IWB45967	138.13	1.3	809.83–820.28	2	2	2	170
	4A	MQTL-YLD8	IWB9592-IWB64680	205.69	1.35	714.43–744.31	2	2	2	510
	4B	MQTL-YLD9	IWA3325-IWB65023	84.87	4.5	443.45–495.60	4	3	3	300
	4D	MQTL-YLD10	IWB24404-Xbarc225	17.66	2.74	1.24–36.44	2	2	2	574
	4D	MQTL-YLD11	wPt-0321-Xbarc288	34.9	0.01	83.24–121.19	3	2	2	286
	5A	MQTL-YLD12	IWB8237-IWA5735	67.51	7	391.54–419.68	2	2	2	176
	5A	MQTL-YLD13	IWB47676-IWB73141	86.12	4.69	442.70–461.49	4	4	4	202
	5B	MQTL-YLD14	wPt-7240-IWB5257	41.47	5.26	7.36–13.42	2	2	2	75
	5B	MQTL-YLD15	IWB8594-IWB13251	75.61	0.84	236.26–244.41	2	2	2	25
	5B	MQTL-YLD16	wPt-3763-IWB74045	140.68	4.79	562.48–589.12	2	2	2	304
	6B	MQTL-YLD17	IWA8165-IWB24239	135.19	2.53	469.07–546.65	4	2	2	443
	6D	MQTL-YLD18	Xbarc173-IWB57751	31.62	1.04	13.03–163.83	4	3	3	1416
	7A	MQTL-YLD19	IWA5489-IWB7435	168.33	3.85	660.55–671.21	4	4	4	133
	7B	MQTL-YLD20	IWB7155-IWB36522	38.72	15.13	0.43–3.33	2	2	2	43
	7B	MQTL-YLD21	IWB35038-IWB13248	78.04	2.87	15.30–21.66	2	2	2	40
	7B	MQTL-YLD22	IWB40563-IWB71926	163.7	2.63	403.56–481.31	2	2	2	418
RL	5B	MQTL-RL1	IWB29709-IWB56759	86.4	2.55	440.51–473.87	3	3	3	299

**Fig. 5.**
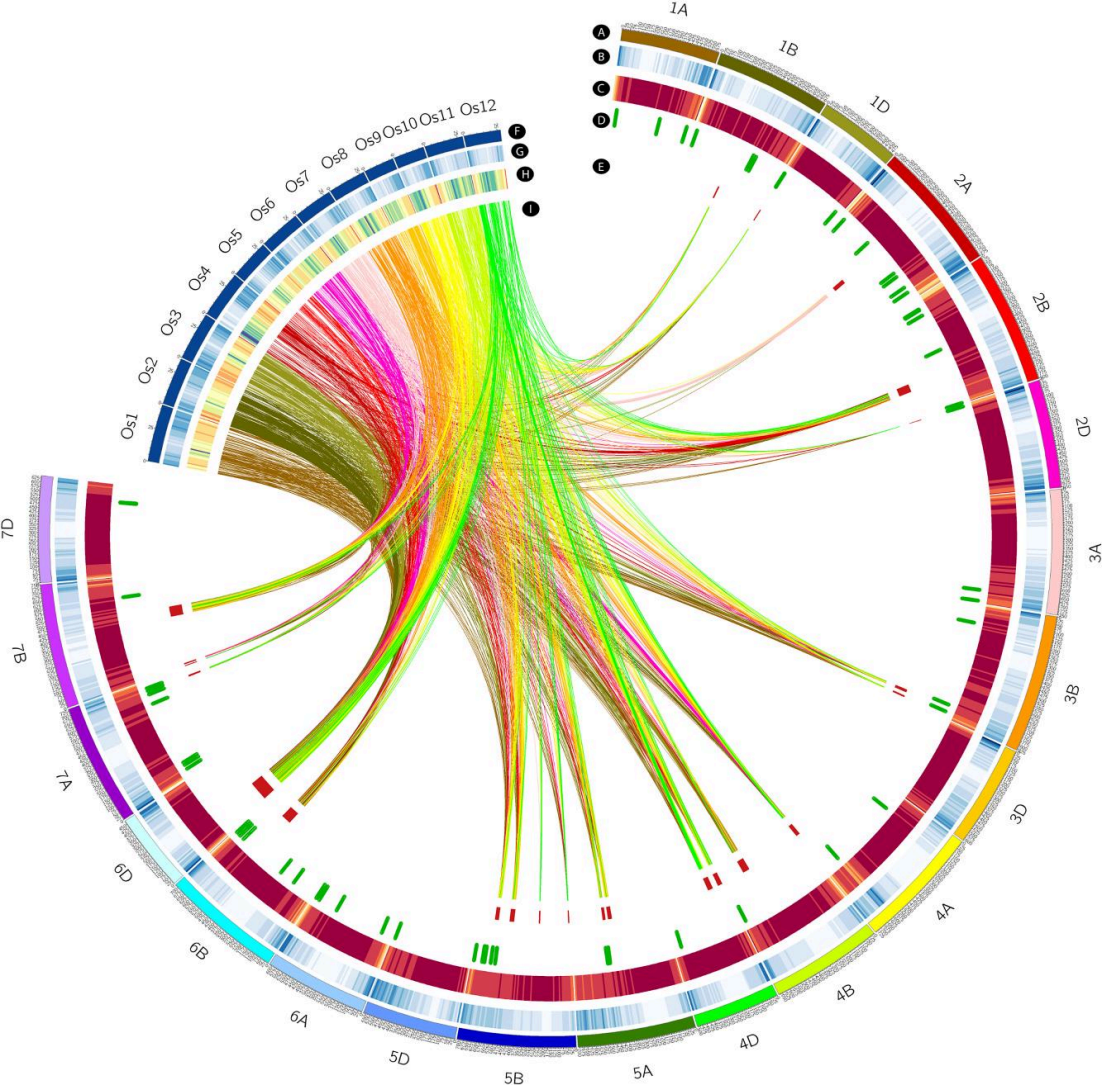
The distribution pattern of a) wheat genome, b) gene density of wheat genome, c) variation density of wheat genome, d) significant GWAS signal on wheat genome, e) MQTLs position on wheat genome, f) rice genome, g) gene density of rice genome h) variation density of rice genome, and i) region of orthologous genes of wheat in rice.

**Fig. 6.**
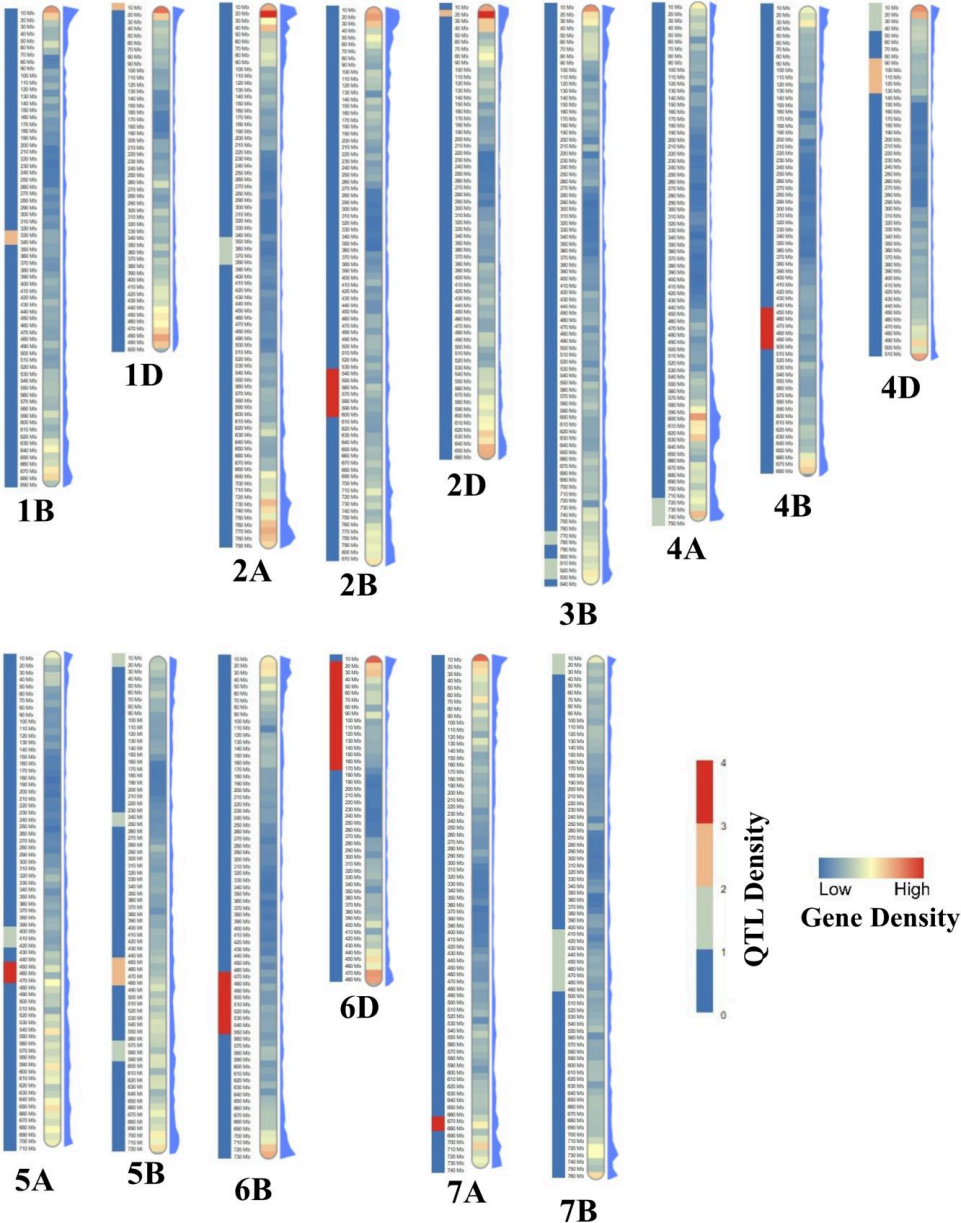
Heatmap of MQTLs for grain yield and RL on the wheat genome based on Mb. The gene and SNP densities of each chromosome are indicated on the chromosome and on the right side of it.

The genomic distribution of MQTLs across the wheat genome was thoroughly assessed which provided us with further valuable information on the position of MQTLs (Supplementary Tables S8 and S9). Gene and SNP densities were obtained from ENsemblPlants and were compared with the genomic position of the identified MQTLs (Figs. 5 and 6). These results indicate that sub-telomeric regions harbor most of the MQTLs which is a pattern similar to what was observed for gene and SNP densities. One of the most valuable benefits of an MQTL analysis is that it can greatly confine the CI of QTLs, improving the chance to identify putative CGs. In our study, the MQTL analysis reduced the average CI of QTLs by 2.63 folds in comparison to the mean of CI of projected QTLs. Three MQTLs including MQTL-YLD1, MQTL-YLD11, and MQTL-YLD15 experienced a great reduction in CIs, with regions spanning less than 1 cM (Table 3). All genes underlying each MQTL region were reported in Supplementary Table S10 as well as their orthologous gene from rice in Supplementary Table S11. Among the annotated genes from the MQTLs, several well-known genes based on their functions in rice were noticed. This includes the rice orthologous *Salt tolerance receptor-like cytoplasmic kinase 1*(*STRK1)* (*TraesCS2B02G407100*), a chloroplast-localized DEAD-box RNA helicases, *OsRH58* (*TraesCS3B02G592900*), *Basic leucine zipper 72 (OsbZIP72)*(*TraesCS5A02G237200*), *AT-HOOK motif nuclear-localized protein 1* (*OsAHL1*) (*TraesCS5B02G129200* and *TraesCS5B02G130400*), *Stress-activated protein kinase 8* (*OsSAPK8*) (*TraesCS5B02G406400*), *Dehydration responsive element-binding 1G,* (*OsDREB1G*) (*TraesCS6B02G268100*), *OsDREB1D* (*TraesCS6D02G173500*), and *dense and erect panicle 3*(*DEP3*) (*TraesCS7A02G464400*) which are located in MQTL-YLD4, MQTL-YLD7, MQTL-YLD13, MQTL-YLD15, MQTL-YLD16, MQTL-YLD17, MQTL-YLD18, and MQTL-YLD19. Further genes and putative CGs are reported in Supplementary Tables S8 and S9. Subsequently, the genes obtaining from all MQTL intervals were compared with the reported differential expressed genes from different transcriptomics studies. A total of 329 genes were detected as a common gene between the RNAseq, MQTLs and GWAS analyses (Supplementary Fig. S2). A KEGG analysis indicated that Nitrogen metabolism, Monoterpenoid biosynthesis, and Cutin, suberine and wax biosynthesis were enriched in our study.

## Discussion

### Phenotypic diversity of seedling traits

The development of high yield and drought-tolerant wheat varieties is undoubtedly one of the most promising targets for wheat breeders across the world due to the significant contributions this crop have to the human diet (Appels *et al*., 2018; Webber *et al*., 2018; Raza *et al*., 2019). In this study, the diversity of Iranian wheat landraces selected from different geographic locations was surveyed based on early vigor related traits and grain production in mature plants. Significant phenotypic diversity was observed for all studied traits and for all drought tolerance indices. A positive and significant correlation was observed between N-YLD (grain yield under normal irrigation) and several early vigor traits such as TFW (*r* = 0.41**), RL (*r* = 0.30**), and GR (*r* = 0.22*). These traits show positive but non-significant correlations with YLD, although the correlation with GR and seedling RL was relatively high. Interestingly, early vigor traits (except for GP) were positively and significantly correlated with two important drought tolerance indices (GMP and STI), highlighting the potential role of good seedling establishment in increasing drought tolerance which is in line with previous studies (Ludwig and Asseng, 2010; Ryan *et al*., 2015; Vukasovic *et al*., 2022).

### GWAS of seedling traits

The phenotypic and SNP datasets were used to explore genetic diversity and to identify loci associated with early vigor traits using genome-wide association mapping. Analyses of population structure identified three semi-isolated clusters of individuals among landraces in both the PCA and variance-covariance (kinship) matrix of individuals. Including principal components and a kinship matrix allows for adequate control of false positives due to population structure (QQ-plots in Figs. 2 and 3). In total, 54 MTAs were identified across all traits under water deficit conditions. The majority of the MTAs were located in the A and B sub-genomes, in line with results from earlier studies in wheat (Mathew *et al*., 2019; Maulana *et al*., 2020; Ahmed *et al*., 2022). The GWAS results then were used to identify putative CGs located in the vicinity of the MTAs. Around three-quarters of all MTAs could be assigned to or near a protein-coding gene. These genes were shown to be involved in a range of biological processes and a GO analysis identified significant enrichments for starch and sucrose metabolism and plant hormone signal transduction.

### Distribution of QTLs and MQTLs

A total of 215 projected QTLs were identified in a literature survey and were used in our MQTL analysis. The uneven distribution of QTLs across chromosomes and sub-genomes of wheat were apparent also in this analysis, where the B sub-genome and in particular chromosome 5B harbored the largest number of QTLs with 101 and 26, respectively. Similarly, and in line with many earlier studies (Zhang *et al*., 2010; Darzi-Ramandi *et al*., 2017; Kumar *et al*., 2020), the D sub-genome contributed the lowest number of QTLs. An MQTL analysis is an approach to identify most stable QTLs, regardless of genetic backgrounds, locations and years and can help reduce the number of stable QTLs and genomic regions controlling traits of interest (Zhu *et al*., 2018; Wang *et al*., 2019; Khahani *et al*., 2019; Khahani *et al*., 2020). In this study, 23 MQTLs were detected that were located across all wheat chromosomes for YLD and RL traits. Chromosomes 5B and 7B harbored the highest number of MQTLs for YL. In comparison with a previous study on yield traits under drought conditions, 6 common MQTLs including MQTL-YLD6, MQTL-YLD8, MQTL-YLD13, MQTL-YLD18, MQTL-YLD19 and MQTL-YLD22 were identified (Liu *et al*., 2020a). Furthermore, 6 common MQTLs were obtained with a previous study under unstressed conditions including MQTL-YLD6, MQTL-YLD8, MQTL-YLD10, MQTL-YLD16, MQTL-YLD19 and MQTL-YLD20 (Yang *et al*., 2021). MQTL-YLD13, MQTL-YLD18 and MQTL-YLD19 had the highest number of QTLs in our study. It is noteworthy to mention that previous investigations have reported that gene density was positively associated with QTL density in maize, barley and rice (Martinez *et al*., 2016; Khahani *et al*., 2019, 2020). Our study reveals that most of MQTLs are located in the sub-telomeric regions where the gene density and densities of SNP are also highest in wheat. This is inconsistent with previous results on the genomic position of MQTLs and gene density in maize, barley, and rice (Martinez *et al*., 2016; Khahani *et al*., 2019, 2020, 2021).

### Identification of putative CGs

An MQTL analysis is a statistical tool that can help to substantially reduce the CI of stable QTLs compared to initial QTLs, allowing us to restrict the genomic regions harboring CGs even further (Bilgerami *et al*., 2020; Khahani *et al*., 2020; Khahani *et al*., 2021; Zheng *et al*., 2021). The accuracy and precision of predicting CGs are therefore higher when QTLs can be mapped with greatly reduced CI intervals (Martinez *et al*., 2016; Khahani *et al*., 2019). The mean CI in our analysis declined by up to 2.63-fold compared to the CIs of QTLs in the original publications. The MQTLs predicting the underlying genes were further analyzed as well as their orthologous genes from rice were identified. Twenty-two MQTLs for YLD trait were detected including three MQTLs on chromosomes 5B and 7B, two MQTLs on chromosomes 3B, 4D, and 5A, one MQTL on chromosomes 1B, 1D, 2A, 2B, 2D, 4A, 4B, 6B, 6D, and 7A. The MQT-YLD4, MQT-YLD9, MQT-YLD13, MQT-YLD17, MQT-YLD18, and MQT-YLD19 accounted for the highest number of projected QTLs and are therefore considered our most stable QTLs. Among CGs within MQTL-YLD4, one of the stable MQTLs with large numbers of projected QTLs harbors the wheat gene *TraesCS2B02G403000* which is homologous to the rice gene *Vacuolar invertase 2* (Os*INV2*). *OsINV2* has a major effect on grain yield by interacting with Os*INV3* and functions by changing sucrose metabolism and grain size (Deng *et al*., 2020). Moreover, the ortholog to the rice gene *STRK1* in wheat, *TraesCS2B02G407100*, has previously been shown to simultaneously improve salt tolerance mechanisms and grain yield (Zhou *et al*., 2018).

In MQTL-YLD7 located on chromosome 3B, genes *TraesCS3B02G592400*, homologous to *Drought-induced* genes in rice such as *OsDi19-5*, *TraesCS3B02G592900*, homologous to rice *OsRH58*, and *TraesCS3B02G594200*, homologous to rice *MODD* (Mediator of *OsbZIP46* deactivation and degradation), were found which are all known to regulate salt and drought tolerance in rice (Tang *et al*., 2016; Nawaz and Kang, 2019; Jing *et al*., 2021). These genes are thus excellent candidates for further analysis on how they might contribute to salt and drought tolerance in wheat. A potential CG in wheat, *TraesCS4B02G208600*, which is located in MQTL-YLD9 interval on chromosome 4B was detected. This gene is orthologous to rice *Salt-and drought-induced ring finger 1* (*OsSDIR1*) which has been shown to enhance drought tolerance compared to wild-type rice when overexpressed (Gao *et al*., 2011). Two other important genes in wheat, *TraesCS4B02G235000*, and *TraesCS4B02G235100*, are orthologous with the rice genes *Similar to rcd one 1c* (*OsSRO1c*) and *drought- and salt-sensitive mutant 3*  **(***DSM3*), respectively. These two genes are key factors in regulating abiotic stresses and in particular salt and drought stresses in rice (Du *et al*., 2011; You *et al*., 2013). Similarly, the wheat genes *TraesCS4D02G013300* and *TraesCS4D02G013400*, located in MQTL-YLD10 on chromosome 4D, are orthologous to rice *CATION/CA2 + EXCHANGER2* (*OsCCX2*), which has been reported to play a role under drought and salt conditions (Yadav *et al*., 2015).

MQTL-YLD13 is located on chromosome 5A and contains a wheat gene, *TraesCS5A02G237200*, that is orthologous to *OsbZIP72*, which is previously shown to have a significant effect on drought tolerance mediated through the abscisic acid pathway (Lu *et al*., 2009). MQTL-YLD15 on chromosome 5B contains two wheat genes, *TraesCS5B02G129200* and *TraesCS5B02G130400*, which are homologous to *OsAHL1* appear to be involved in drought resistance (Zhou *et al*., 2016). A heat gene orthologous to the rice gene *gibberellin-stimulated transcript-related gene 1* (*OsGASR1*) is located at MQTL-YLD16 on chromosome 5B. The rice ortholog has been reported to regulate cellular mechanisms against salt stress (Lee *et al*., 2017). Another CG in the same MQTL, *TraesCS5B02G406400*, is orthologous *SAPK8* which regulates drought tolerance positively in rice (Zhong *et al*., 2020). Among the CGs identified in MQTL-YLD17 on chromosome 6B, several potentially important genes were found. For example, *TraesCS6B02G265000*, is homologous to rice *Salt Intolerance 1* (*SIT1*) and plays a regulatory role under salt stress which might improve the defense mechanism (Zhao *et al*., 2019b). Similarly, the rice ortholog of *TraesCS6B02G286500*, *DROUGHT HYPERSENSITIVE* (*DHS*), contributes to drought stress (Wang *et al*., 2017).

MQTL-YLD18 on chromosome 6D contains the largest number of CGs, including *TraesCS6D02G066700*, *TraesCS6D02G083500*, *TraesCS6D02G109800*, *TraesCS6D02G167500, TraesCS6D02G171100* and *TraesCS6D02G173500* which are orthologous to *LATERAL ROOTLESS2* (*LRT2*), *STRESS tolerance and GRAIN_LENGTH* (*OsSGL*), *Protein phosphatase18* (*OsPP18*), stress repressive zinc finger protein 1 (*SRZ1*)*, gamma-ray induced Leucine-rich repeat receptor-like kinase* (*OsGIRL1*) and *OsDREB1D* in rice. These genes are good candidates to further evaluation in wheat as they have important functions under abiotic stresses in rice (Lee *et al*., 2013; Park *et al*., 2014; You *et al*., 2014; Kumari *et al*., 2015; Wang *et al*., 2016). Finally, the wheat genes *TraesCS7A02G464400* and *TraesCS7A02G464800* are located at MQTL-YLD19 on chromosome 7A and are orthologous with rice *DEP3* and*Oryza sativa Yellow37* (*ONAC011*, *OsY37*). In rice, these genes play a substantial role in improving grain yield and drought tolerance (Qiao *et al*., 2011; Fang *et al*., 2014). Furthermore, a comparison of common genes between RNAseq studies with genes retrieved from MQTLs revealed that Nitrogen metabolism, Monoterpenoid biosynthesis, and Cutin, suberine and wax biosynthesis were enriched based on the KEGG enrichment analysis. Water deficit stress affects the metabolism of nitrogen in plants by decreasing the total N content (Tang *et al*., 2020). Cutin and suberine biosynthesis play a regulatory role in providing mechanisms against water deprivation in plants (Xue *et al*., 2017; Ayaz *et al*., 2021).

### Conclusion

We implemented a GWAS approach to identify loci associated with early vigor in wheat under simulated water deficit and grain yield under drought condition in the field. Fifty-four MTAs were identified that are located across the wheat genome but with the highest number found in the B sub-genome. Further analyses are required to confirm many of the genomic regions that have been identified in this study. An MQTL analysis was then performed to further expand our approach to identify genomic regions associated with early vigor traits and grain yield and to validate identified MTAs in the current study. Most of the MQTLs detected are in sub-telomeric regions and coincides with regions of high gene and SNP densities. MQTL regions MQT-YLD4, MQT-YLD9, MQT-YLD13, MQT-YLD17, MQT-YLD18, MQT-YLD19, and MQTL-RL1 contained the highest number of projected QTLs and were therefore regarded as the most reliable and stable QTLs under different environmental conditions. These MQTLs facilitate the identification of CGs underlying at each MQTL interval due to the reduced CI associated with MQTLs. Moreover, comparing results from the GWAS, MQTL and RNA-seq studies identified a common gene, *TraesCS4A02G485800*, which is homologous to the rice gene *OsVIN3,* member of vacuolar invertases. This gene plays a major role in sugar metabolism and in mediating wheat grain size. Additionally, this gene has been reported to act as a regulator under water stressed conditions in previous using RNAseq studies (Deng *et al*., 2020). These findings provide important information on the genetic basis of seedling vigor traits under simulated water deficit and grain yield under drought stress and set the foundation for future investigations into adaptation to drought conditions in wheat varieties.

## Supplementary Material

jkac320_Supplementary_Data

## Data Availability

Supplemental files are available in the following link: https://doi.org/10.6084/m9.figshare.21197440.v1. Supplementary Fig. S1: The number and distribution of initial QTLs and MQTLs for investigated traits. Supplementary Fig. S2: Comparison of GWAS, MTQL and RNA-seq studies in wheat under simulated water deficit and drought condition. Supplementary Table S1: Information of 100 Iranian wheat landraces and four check varieties evaluated in this study. Supplementary Table S2: The germination parameters of 20 selected accessions under simulated water deficit condition in the pilot study. Supplementary Table S3: Phenotypic datasets from simulated water deficit condition and field trial. Supplementary Table S4: Drought tolerance indices used for investigation of Iranian wheat landraces. Supplementary Table S5: Summary of QTL populations used for meta-QTL analysis of grain yield, root length, shoot length, germination rate, total dry weight, and total fresh weight in wheat under water deficit condition. Supplementary Table S6: Distribution of SNP markers and indices of genetic diversity across the wheat genome. Supplementary Table S7: Summary of QTL populations used for meta-QTL analysis of grain yield, root length, shoot length, germination rate, total dry weight and total fresh weight in wheat under water deficit condition. Supplementary Table S8: Description of significant MTAs with germination and grain yield of Iranian wheat landraces exposed to water-deficit condition. Supplementary Table S9: KEGG orthology-based annotation for regions surrounding GWAS significant. Supplementary Table S10: Genes underlying each MQTL region in the wheat genome and their description. Supplementary Table S11: Orthologous gene from rice of the identified wheat genes within each MQTL.

## Notes

*Communicating Editor: E. Akhunov*
