# Supplementary material for: Genome-wide association study to identify genomic loci associated with early vigor in bread wheat under simulated water deficit complemented with quantitative trait loci meta-analysis
Source: G3 (Bethesda). 2022 Dec 2;13(2):jkac320. doi: 10.1093/g3journal/jkac320 (PMC10248217; doi:10.1093/g3journal/jkac320)
Supplement: jkac320_Supplementary_Data [file jkac320_supplementary_data.zip › Supplementary Figures S1 and S2.docx]

Fig. S1. The number and distribution of initial QTLs and MQTLs for investigated traits under water deficit condition. A) The number of QTLs and MQTLs on different chromosomes of wheat. B) The distribution of QTLs for different traits on each chromosome of wheat


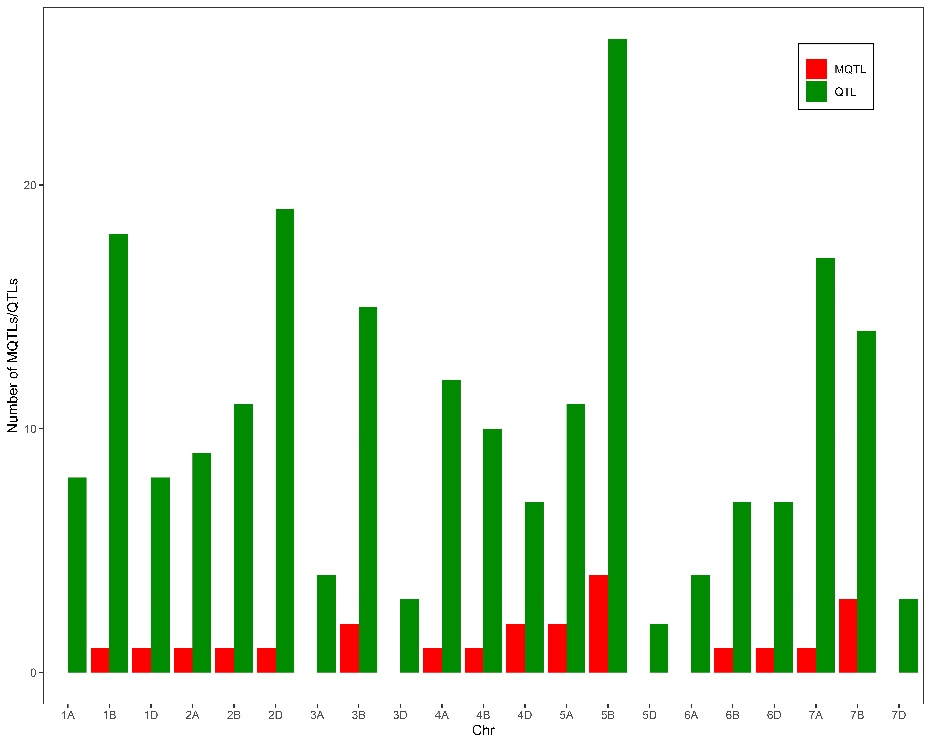

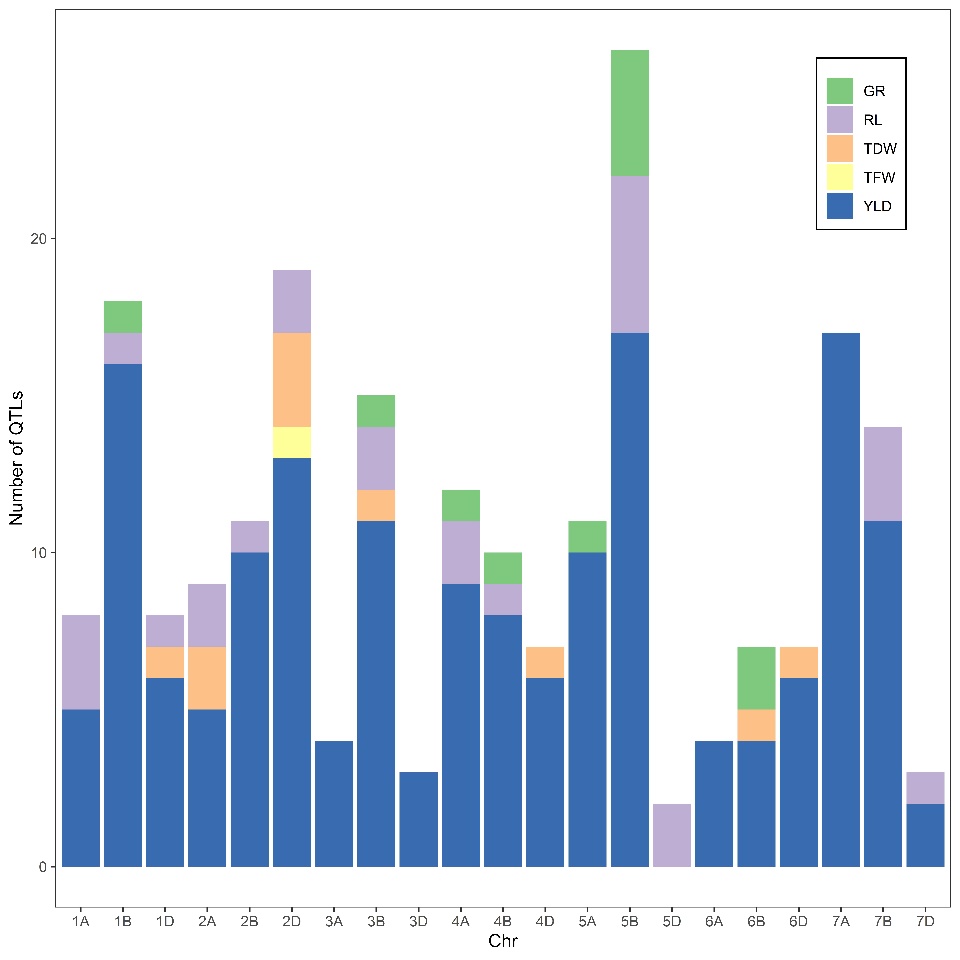


a.

b.


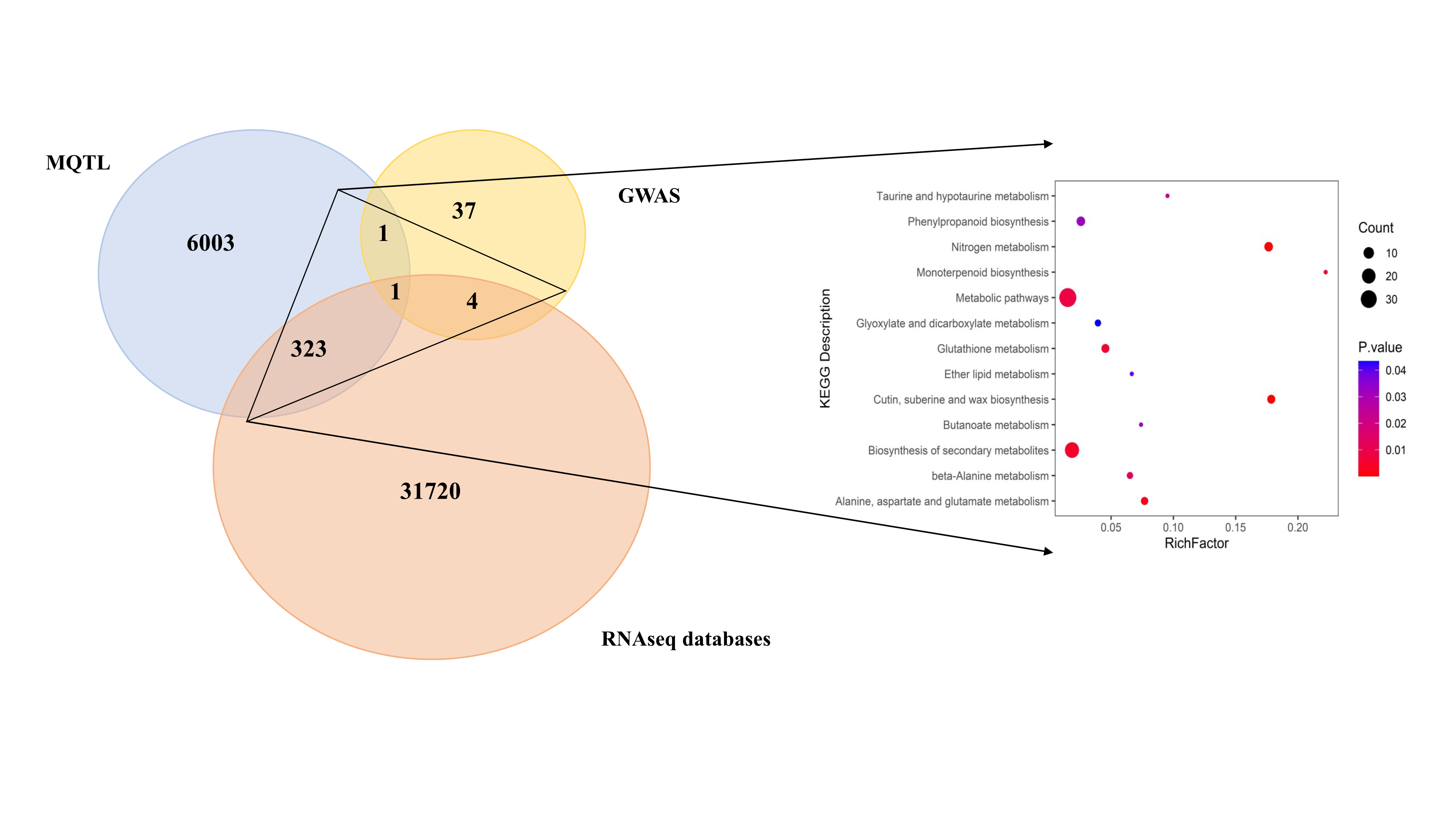
Fig. S2. Comparison of GWAS, MTQL and RNA-seq studies in wheat under simulated water deficit and drought condition
