# Supplementary material for: Genome-wide association study to identify genomic loci associated with early vigor in bread wheat under simulated water deficit complemented with quantitative trait loci meta-analysis
Source: G3 (Bethesda). 2022 Dec 2;13(2):jkac320. doi: 10.1093/g3journal/jkac320 (PMC10248217; doi:10.1093/g3journal/jkac320)
Supplement: jkac320_Supplementary_Data [file jkac320_supplementary_data.zip › Supplementary Tables S2, S4, S5, S6, S7, S8, S9.docx]

Table S2. The germination parameters of 20 selected accessions under simulated water deficit condition in the pilot study

| Treatments |  | Total Germination  (%) | Root length  (cm) | Shoot length  (cm) | Root weight  (g) | Shoot weight  (g) |
| --- | --- | --- | --- | --- | --- | --- |
| Control | No PEG | 99+5.8 a | 13.21+2.51 a | 15.95+2.77 a | 0.27+0.116 a | 0.64+0.18 a |
| Simulated water deficit | -4 (bars) | 97+5.5 ab | 11.26+2.41 bc | 11.14+1.91 b | 0.18+0.06 b | 0.34+0.08 b |
|  | -6 (bars) | 95+10.06 ab | 12+2.05 b | 8.29+1.89 c | 0.17+0.05 bc | 0.28+0.08 c |
|  | -8 (bars) | 95+9.3 ab | 10.62+2.32 c | 5.60+1.44 d | 0.15+0.08 cd | 0.21+0.06 d |
|  | -10 (bars) | 93+9.9 ab | 9.24+2.14 d | 3.25+1.11 e | 0.13+0.08 d | 0.12+0.05 e |
|  | -12 (bars) | 73+20.34 b | 5.76+2.26 e | 0.78+0.67 f | 0.07+0.04 e | 0.024+0.01 f |

Table S4. Drought tolerance indices used for investigation of Iranian wheat landraces

| Index | Abbreviation | Equation | Reference |
| --- | --- | --- | --- |
| Geometric mean productivity | GMP |  | (Mardeh et al. 2006) |
| Stress tolerance index | STI |  | (Fernandez 1993) |

: Performance under normal conditions; : Performance under stress conditions; : Mean performance of the genotypes under normal conditions.

Table S5. Summary of QTL populations used for meta-QTL analysis of grain yield, root length, shoot length, germination rate, total dry weight and total fresh weight in wheat under water deficit condition

| Ref No. | Number of QTL Population(s) | Parents of Population | Population Type | Population Size | Trait(s) | Reference |
| --- | --- | --- | --- | --- | --- | --- |
| 1 | 1 | Dharwar Dry × Sitta | RIL | 140 | YLD | (Kirigwi et al., 2007) |
| 2 | 1 | W7984 × Opata 85 | RIL | 114 | RL, SL | (Landjeva et al., 2008) |
| 3 | 1 | Kofa × Svevo | RIL | 249 | YLD | (Maccaferri et al., 2008) |
| 4 | 1 | Seri M82 × Babax | RIL | 194 | YLD | (Mathews et al., 2008) |
| 5 | 1 | Langdon × G18-16 | RIL | 152 | YLD | (Peleg et al., 2009) |
| 6 | 1 | Seri M82 × Babax | RIL | 194 | YLD | (McIntyre et al., 2010) |
| 7 | 1 | Seri M82 × Babax | RIL | 167 | YLD | (Pinto et al., 2010) |
| 8 | 1 | OsteGata × Massara-1 | F2 | 151 | YLD | (Golabadi et al., 2011) |
| 9 | 1 | Hanxuan 10 × Lumai 14 | DH | 150 | YLD | (Wu et al., 2011) |
| 10 | 1 | Dharwar Dry × Sitta | RIL | 122 | YLD | (Alexander et al., 2012) |
| 11 | 1 | RAC875 × Kukri | DH | 255 | YLD | (Bennett et al., 2012a) |
| 12 | 1 | RAC875 × Kukri | DH | 260 | YLD | (Bennett et al., 2012b) |
| 13 | 1 | RAC875 × Kukri | DH | 368 | YLD | (Bonneau et al., 2012) |
| 14 | 1 | Cranbrook × Halberd | DH | 168 | TDW | (Borras-Gelonch et al., 2012) |
| 15 | 1 | Devon × Syn084 | BC | 177 | RL | (Ibrahim et al., 2012a) |
| 16 | 1 | Triso × Syn084 | BC | 223 | YLD | (Ibrahim et al., 2012b) |
| 17 | 1 | C306 × HUW206 | RIL | 104 | YLD | (Kumar et al., 2012) |
| 18 | 1 | Hanxuan 10 × Lumai | DH | 150 | YLD | (Wu et al., 2012) |
| 19 | 1 | Excalibur × Kukri | DH | 233 | YLD | (Hill et al., 2013) |
| 20 | 1 | Hanxuan10 × Lumai 14 | DH | 150 | YLD, RL | (Liu et al., 2013) |
| 21 | 1 | Seri M82 × Babax | RIL | 165 | YLD | (Lopes et al., 2013) |
| 22 | 2 | Weimai 8 × Luohan 2 | RIL | 229 | RL, TDW | (Zhang et al., 2013) |
|  |  | Weimai 8 × Yannong 9 | RIL | 302 | RL, TFW, TDW |  |
| 23 | 1 | CS × SQ1 | DH | 90 | GR, RL | (Czyczyto-Mysza et al., 2014) |

BC= Backcross, DH= Double Haploids, RIL= Recombinant Inbred Lines. YLD= Yield, RL=Root Length, SL= Shoot Length, GR= Germination Traits, TDW= Total Dry Weight, TFW= Total Fresh Weight

Table S5. Continued.

| Ref No. | Number of QTL Population(s) | Parents of Population | Population Type | Population Size | Trait(s) | Reference |
| --- | --- | --- | --- | --- | --- | --- |
| 24 | 1 | Drysdale × Gladius | RIL | 205 | YLD | (Maphosa et al., 2014) |
| 25 | 1 | McNeal × Thatcher | RIL | 160 | YLD | (Sherman et al., 2014) |
| 26 | 1 | WL711 × C306 | RIL | 206 | YLD | (Shukla et al., 2014) |
| 27 | 1 | Rio Blanco × IDO444 | RIL | 159 | YLD | (Zhang et al., 2014a) |
| 28 | 3 | Weimai 8 × Luohan 2 | RIL | 179 | RL | (Zhang et al., 2014b) |
|  |  | Weimai 8 × Yannong 9 | RIL | 175 | RL |  |
|  |  | Weimai 8 × Jimai 20 | RIL | 172 | RL, TDW |  |
| 29 | 1 | Excalibur × Kukri | DH | 233 | YLD | (Hill et al., 2015) |
| 30 | 1 | Jingdong8 × Aikang58 | RIL | 210 | YLD | (Li et al., 2015) |
| 31 | 1 | Culter × AC Barrie | RIL | 158 | YLD | (Perez-Lara et al., 2016) |
| 32 | 1 | CO960293 × TAM 111 | RIL | 217 | YLD | (Assanga et al., 2017) |
| 33 | 1 | W7984 × Opata 85 | RIL | 104 | RL | (Ayalew et al., 2017) |
| 34 | 1 | Excalibur × Kukri | DH | 192 | YLD, GR | (Gahlaut et al., 2017) |
| 35 | 1 | Seri M82 × Babax | RIL | 167 | YLD | (Tahmasebi et al., 2017) |
| 36 | 1 | Chuan 35050 × Shannong 483 | RIL | 131 | YLD | (Xu et al., 2017) |
| 37 | 1 | W7984 × Opata 85 | RIL | 104 | TDW | (Ayalew et al., 2018) |
| 38 | 1 | CO940610 × Platte | DH | 185 | YLD, TDW | (El-Feki et al., 2018) |
| 39 | 1 | W7984 × Opata 85 | RIL | 209 | RL, SL, TDW | (Khalid et al., 2018) |
| 40 | 1 | HD2733 × HI1500 | BC | 516 | YLD | (Rai et al., 2018) |
| 41 | 1 | SYN-D × Weebill | RIL | 276 | YLD | (Liu et al., 2019a) |
| 42 | 1 | Seri × Babax | RIL | 156 | YLD | (Liu et al., 2019b) |
| 43 | 1 | Omrabi5 × Belikh2 | RIL | 114 | YLD, GR | (Arif et al., 2020) |
| 44 | 1 | Vida × MTHW0202 | RIL | 155 | YLD | (Cook et al., 2020) |
| 45 | 1 | RAC875 × Kukri | DH | 220 | YLD, RL | (Salarpour et al., 2020) |
| 46 | 1 | TAM 112 × TAM 111 | RIL | 124 | YLD | (Yang et al., 2020) |
| 47 | 1 | Roshan × Superhead#2 | RIL | 186 | YLD | (Zandipour et al., 2020) |
| 48 | 1 | Avalon × Cadenza | DH | 201 | YLD | (Amalova et al., 2021) |
| 49 | 1 | LDN × WEW | RIL | 150 | YLD, TDW | (Fatiukha et al., 2021) |

BC= Backcross, DH= Double Haploids, RIL= Recombinant Inbred Lines. YLD= Yield, RL=Root Length, SL= Shoot Length, GR= Germination Traits, TDW= Total Dry Weight, TFW= Total Fresh Weight

Table S6. Distribution of SNP markers and indices of genetic diversity across the wheat genome

| Chromosome | Length (Mb) | No. SNP | Density | MAF | Gene  Diversity | Heterozygosity | PIC | A↔G | T↔C | A↔T | A↔C | G↔T | G↔C | Ts | Tv | Ts/Tv |
| --- | --- | --- | --- | --- | --- | --- | --- | --- | --- | --- | --- | --- | --- | --- | --- | --- |
| 1A | 594.0 | 540 | 0.9091 | 0.1981 | 0.2796 | 0.0526 | 0.2295 | 187 | 187 | 20 | 41 | 37 | 68 | 374 | 166 | 2.2530 |
| 2A | 780.7 | 568 | 0.7276 | 0.2248 | 0.3026 | 0.0571 | 0.2448 | 215 | 170 | 32 | 44 | 41 | 66 | 385 | 183 | 2.1038 |
| 3A | 750.8 | 548 | 0.7299 | 0.1640 | 0.2355 | 0.0496 | 0.1950 | 196 | 173 | 35 | 44 | 37 | 63 | 369 | 179 | 2.0615 |
| 4A | 744.5 | 440 | 0.5910 | 0.1810 | 0.2517 | 0.0509 | 0.2074 | 147 | 127 | 27 | 38 | 32 | 69 | 274 | 166 | 1.6506 |
| 5A | 709.7 | 431 | 0.6073 | 0.1865 | 0.2702 | 0.0520 | 0.2231 | 140 | 126 | 31 | 33 | 43 | 58 | 266 | 165 | 1.6121 |
| 6A | 618.0 | 411 | 0.6650 | 0.2161 | 0.3011 | 0.0551 | 0.2454 | 141 | 123 | 19 | 35 | 31 | 62 | 264 | 147 | 1.7959 |
| 7A | 736.6 | 732 | 0.9938 | 0.1941 | 0.2734 | 0.0476 | 0.2237 | 239 | 223 | 35 | 76 | 52 | 107 | 462 | 270 | 1.7111 |
| A genome | 4934.5 | 3670 | 0.7437 | 0.1950 | 0.2733 | 0.0519 | 0.2239 | 1265 | 1129 | 199 | 311 | 273 | 493 | 2394 | 1276 | 1.8762 |
| 1B | 689.4 | 652 | 0.9457 | 0.2055 | 0.2919 | 0.0551 | 0.2390 | 226 | 193 | 43 | 48 | 45 | 97 | 419 | 233 | 1.7983 |
| 2B | 801.2 | 835 | 1.0422 | 0.2091 | 0.2850 | 0.0532 | 0.2308 | 276 | 236 | 38 | 72 | 71 | 142 | 512 | 323 | 1.5851 |
| 3B | 830.3 | 799 | 0.9623 | 0.1909 | 0.2733 | 0.0536 | 0.2246 | 260 | 264 | 43 | 71 | 59 | 102 | 524 | 275 | 1.9055 |
| 4B | 673.4 | 314 | 0.4663 | 0.1340 | 0.1904 | 0.0424 | 0.1584 | 96 | 98 | 15 | 23 | 31 | 51 | 194 | 120 | 1.6167 |
| 5B | 713.0 | 694 | 0.9734 | 0.2079 | 0.2900 | 0.0550 | 0.2368 | 217 | 212 | 45 | 59 | 60 | 101 | 429 | 265 | 1.6189 |
| 6B | 720.9 | 663 | 0.9197 | 0.2046 | 0.2907 | 0.0540 | 0.2380 | 238 | 196 | 30 | 49 | 53 | 97 | 434 | 229 | 1.8952 |
| 7B | 750.6 | 649 | 0.8646 | 0.1705 | 0.2526 | 0.0507 | 0.2100 | 211 | 214 | 23 | 60 | 37 | 104 | 425 | 224 | 1.8973 |
| B genome | 5179.0 | 4606 | 0.8894 | 0.1940 | 0.2745 | 0.0528 | 0.2250 | 1524 | 1413 | 237 | 382 | 356 | 694 | 2937 | 1669 | 1.7597 |
| 1D | 495.4 | 310 | 0.6258 | 0.1953 | 0.2775 | 0.0487 | 0.2291 | 94 | 81 | 23 | 34 | 23 | 55 | 175 | 135 | 1.2963 |
| 2D | 651.6 | 385 | 0.5909 | 0.1455 | 0.2185 | 0.0454 | 0.1851 | 123 | 110 | 23 | 32 | 26 | 71 | 233 | 152 | 1.5329 |
| 3D | 615.4 | 193 | 0.3136 | 0.1410 | 0.2182 | 0.0370 | 0.1870 | 65 | 40 | 11 | 23 | 24 | 30 | 105 | 88 | 1.1932 |
| 4D | 509.6 | 84 | 0.1648 | 0.2250 | 0.2967 | 0.0509 | 0.2389 | 28 | 26 | 7 | 5 | 9 | 9 | 54 | 30 | 1.8000 |
| 5D | 566.0 | 179 | 0.3163 | 0.1481 | 0.2307 | 0.0453 | 0.1962 | 46 | 60 | 13 | 16 | 14 | 30 | 106 | 73 | 1.4521 |
| 6D | 473.5 | 203 | 0.4287 | 0.2331 | 0.3149 | 0.0537 | 0.2547 | 65 | 53 | 14 | 23 | 16 | 32 | 118 | 85 | 1.3882 |
| 7D | 638.6 | 269 | 0.4212 | 0.1989 | 0.2785 | 0.0528 | 0.2274 | 80 | 75 | 17 | 22 | 28 | 47 | 155 | 114 | 1.3596 |
| D genome | 3950.4 | 1623 | 0.4108 | 0.1787 | 0.2571 | 0.0476 | 0.2134 | 501 | 445 | 108 | 155 | 140 | 274 | 946 | 677 | 1.3973 |
| Unknown | - | 1039 | - | 0.1829 | 0.2634 | 0.0530 | 0.2176 | 338 | 320 | 55 | 81 | 83 | 162 | 658 | 381 | 1.7270 |
| The whole genome | 14063.9 | 10938 | 0.7777 | 0.1910 | 0.2705 | 0.0518 | 0.2222 | 3628 | 3307 | 599 | 929 | 852 | 1623 | 6935 | 4003 | 1.7325 |

Table S7. A summary of observed LD among SNP marker pairs and the number of significant marker pairs per chromosome and genome

| Chromosome | TNSP | Distance (cM) | r2 | NSSP |
| --- | --- | --- | --- | --- |
| 1A | 25725 | 0.5259 | 0.099162 | 4083 (15.87%) |
| 2A | 28400 | 0.4052 | 0.127385 | 5922 (20.85%) |
| 3A | 27400 | 0.7574 | 0.084587 | 2625 (9.58%) |
| 4A | 22000 | 0.6839 | 0.122501 | 3082 (14.01%) |
| 5A | 21550 | 0.6740 | 0.092404 | 3186 (14.78%) |
| 6A | 20550 | 0.6424 | 0.12255 | 4490 (21.85%) |
| 7A | 36600 | 0.4770 | 0.120252 | 5844 (15.97%) |
| A genome | 182225 | 0.5818 | 0.110261 | 29232 (16.04%) |
| 1B | 32600 | 0.3822 | 0.109502 | 6315 (19.37%) |
| 2B | 41750 | 0.3381 | 0.107019 | 7095 (16.99%) |
| 3B | 39950 | 0.3774 | 0.109594 | 6767 (16.94%) |
| 4B | 15700 | 0.7025 | 0.063016 | 854 (5.44%) |
| 5B | 34700 | 0.5470 | 0.082284 | 4267 (12.3%) |
| 6B | 33150 | 0.3780 | 0.08523 | 4825 (14.56%) |
| 7B | 32450 | 0.4081 | 0.091475 | 4062 (12.52%) |
| B genome | 230300 | 0.4231 | 0.095764 | 34185 (14.84%) |
| 1D | 15500 | 1.0468 | 0.128434 | 3371 (21.75%) |
| 2D | 19250 | 0.5784 | 0.180985 | 3694 (19.19%) |
| 3D | 9650 | 1.7579 | 0.101119 | 1258 (13.04%) |
| 4D | 4200 | 2.2498 | 0.121702 | 657 (15.64%) |
| 5D | 8950 | 2.5787 | 0.078479 | 918 (10.26%) |
| 6D | 10150 | 1.6381 | 0.087983 | 1438 (14.17%) |
| 7D | 13450 | 1.5882 | 0.102884 | 2029 (15.09%) |
| D genome | 81150 | 1.4151 | 0.1225 | 13365 (16.47%) |
| The whole genome | 493675 | 0.6447 | 0.10551 | 76782 (15.55%) |

Table S8. Description of significant MTAs with germination and grain yield of Iranian wheat landraces exposed to water-deficit condition

| Trait | Marker | Sequence | Gene | Gene Position | Molecular function | Biological process | Cellular component |
| --- | --- | --- | --- | --- | --- | --- | --- |
| TDW | rs15067 | TGCAGCACGCTCACTGGCAGCGCCGCCACCACCAGGAGACCACCGTCGCACCACCTTCACCCGA | TraesCS2B02G109300 | Chromosome 2B: 70,795,643-70,797,013 | protein binding | intracellular protein transport | intracellular anatomical structure |
|  | rs60320 | TGCAGTGCAAAAGTCTGCGGCCGCAAAAGCACAAGCGGACATAAGGCCAACTCCACCGCGCGAC | TraesCS2A02G095000 | Chromosome 2A: 48,456,275-48,456,707 | - | - | - |
|  | rs14242 | TGCAGCACCCCAATGATGCAAGCTGGCTAGAATCCTTCCTGGCCCGATCCTGCACGAAGCTAGC | TraesCS3B02G521200 | Chromosome 3B: 764,528,492-764,529,396 | - | - | - |
|  | rs48905 | TGCAGGCTCCTCTCGATGGAGACTGGAGAGCGCCGCCGACGACTTCCGCGCCACGCGCAGCCGC | TraesCS7A02G076700 | Chromosome 7A: 42,001,695-42,005,511 |  | regulation of transcription, DNA-templated | nucleus |
| TFW | rs52171 | TGCAGGTAACCAACGGCCCAGTGTAGCGCGGGCGCCCCCCTGGGTCGATCGGTCAGGTGGTAAC | TraesCS2A02G323200 | Chromosome 2A: 553,278,305-553,280,315 | transcription coregulator activity, histone acetyltransferase activity, protein binding, zinc ion binding | regulation of transcription, DNA-templated | nucleus |
| GR | rs17308 | TGCAGCAGCGGTGGTCGTCGCCTTGTATGTGTCGAGCTTCCAACGTGCAGGTGTGCTTCACTAA | TraesCS1D02G150000 | Chromosome 1D: 206,501,556-206,513,457 | oxidoreductase activity, acting on CH-OH group of donors, long-chain-alcohol oxidase activity, flavin adenine dinucleotide binding | oxidation-reduction process | membrane |
|  | rs62908 | TGCAGTTCACGAGCGAGAGGCTCCCACGACAAACACGAGGGAGACTCAGCGATGGATAGCTCCG | TraesCS4B02G269300 | Chromosome 4B: 544,295,251-544,297,535 | - | - | - |

Table S8. Continued.

| Trait | Marker | Sequence | Gene | Gene Position | Molecular function | Biological process | Cellular component |
| --- | --- | --- | --- | --- | --- | --- | --- |
| GR | rs46239 | TGCAGGCAGATAGTGTTCTCTGTCGACGTGGACACCGCGGTCAATGTCGGCTTGGTACCGCTGC | TraesCS6A02G381900 , TraesCS6D02G366600, TraesCS6B02G421100 | Chromosome 6A: 601,230,996-601,233,757 and Chromosome 6D: 454,939,484-454,941,391 and Chromosome 6B: 692,036,461-692,038,525 | protein binding | - | - |
|  | rs46764 | TGCAGGCCACCAGCCATTCTCCTAATGGCAAAGCCAAATTTCCATGGCCACTTATGGTCTGGCT | TraesCS7B02G342200 | Chromosome 7B: 597,238,265-597,241,423 | cis-regulatory region sequence-specific DNA binding, protein binding, | positive regulation of seed germination, histone H4-R3 methylation | nucleus |
| NGP | rs58619 | TGCAGTCCGCCCCGTTGTCGCACGCGTAGTCCAGCGCCTCCTGCAAGGCCGCATCCCCGACCGC | TraesCS1B02G454500 | Chromosome 1B: 670,158,858-670,161,009 | hydrolase activity, hydrolyzing O-glycosyl compounds | carbohydrate metabolic process | - |
|  | rs39056 | TGCAGCTGCGGGCACATGGAGACCACTCCTGTGTAGGCCATCATCGGCTGGCTGAAGTCCTGAA | TraesCS4A02G030000 | Chromosome 4A: 22,082,648-22,083,721 | - | - | - |
|  | rs29630 | TGCAGCGCAGCGGACAGGCGGACGCAGCGGTGGTGCGTCTGGCGCCAGGTGAAGGCGGTGCCAC | TraesCS5B02G359300 | Chromosome 5B: 539,247,483-539,249,676 | catalytic activity | - | - |
|  | rs20378 | TGCAGCATTTCCCCCGATACAAAACTCAAATCGCAGGCCAAAAAGGGGCGGGGCGGCGCACGCA | TraesCS7A02G251400 | Chromosome 7A: 235,460,629-235,468,417 | catalytic activity, hydrolase activity, hydrolyzing O-glycosyl compounds, hydrolase activity, hydrolase activity, acting on glycosyl bonds, isoamylase activity | carbohydrate metabolic process, glycogen metabolic process, metabolic process, amylopectin biosynthetic process, starch biosynthetic process | chloroplast isoamylase complex |

Table S8. Continued.

| Trait | Marker | Sequence | Gene | Gene Position | Molecular function | Biological process | Cellular component |
| --- | --- | --- | --- | --- | --- | --- | --- |
| NGP | rs5221 | TGCAGAGAAGCAAAGCAACGCTAGTCACGCTTCCGTTTTTGACCATCAAACAGGGAAAGAGAGC | TraesCS7D02G400300 | Chromosome 7D: 517,218,864-517,219,785 | - | - | - |
| RL | rs5997 | TGCAGAGCCCCACAACCATCAAATCCGAGGTGTCGGAGGGAGGTAAGGAGCTGAAGCGCCTTCT | TraesCS1B02G036800 | Chromosome 1B: 17,564,886-17,567,432 | ADP binding | - | - |
|  | rs26144 | TGCAGCCTAAACTATAGCAGTTTGCAATCTAGGGTGCTATGCATCAAGAAGTAGCAGGGATGGC | TraesCS3A02G414700 | Chromosome 3A: 658,238,348-658,244,566 | - | snRNA processing | integrator complex |
|  | rs3564 | TGCAGACATGCAACACGTCCATGAAAGCTACCATCTGGTTCTCAGCTAACTGTATAAGGGAAAA | TraesCS3A02G416700 | Chromosome 3A: 659,026,954-659,038,375 | N-acetyltransferase activity | - | - |
|  | rs42327 | TGCAGGAACGAAGTGATCTTGACGAGGTCCTCCGCCTCCTCAGGACGGGCAGAGTAGGTGTCGA | TraesCS3B02G495000 | Chromosome 3B: 739,223,550-739,225,193 | protein binding | - | - |
|  | rs6172 | TGCAGAGCGTCGACCCGCACGGTACAATGGCGCAGCCGCAGCGACTGGAGCCGCGGGAACGCTA | TraesCS3D02G448500 | Chromosome 3D: 556,895,042-556,897,045 | protein binding | - | - |
|  | rs58548 | TGCAGTCCGATCGATCAGGCCCTCGATCTCGAGGTGGCCGTGGTGATCGCCGTGGCGATGCTCA | TraesCS5B02G374800 | Chromosome 5B: 551,805,188-551,808,022 | structural constituent of ribosome, 5S rRNA binding | translation | ribosome |
|  | rs48670 | TGCAGGCTAAGGCCGTCTGGGTCCATCGTCGTCAACCATCTTCCTAAGAGCTCGGGCAGGAGGA | TraesCS6A02G036300 | Chromosome 6A: 17,897,330-17,904,083 | protein kinase activity, ATP binding | protein phosphorylation | - |

Table S8. Continued.

| Trait | Marker | Sequence | Gene | Gene Position | Molecular function | Biological process | Cellular component |
| --- | --- | --- | --- | --- | --- | --- | --- |
| SL | rs41429 | TGCAGCTTGGAACTGTAGAGGAGGCAGATGGCGCTCCTCGTCATTGCCCACCAGGGAGGCCATA | TraesCS1B02G041900 | Chromosome 1B: 21,804,422-21,806,308 | - | - | - |
|  | rs3536 | TGCAGACATCCTCAGAGTGAATTAACAGCCTGATCTCTCGGCGGCCTCGGAGGGACGAGCAACT | TraesCS5B02G356700 | Chromosome 5B: 536,322,671-536,326,028 | - | transmembrane transport | integral component of membrane |
|  | rs30730 | TGCAGCGCGCTTCCAAGCGACGACTGGTTCATTGAAGTCGCGCAAGACAGCATCAGCAGGATCA | TraesCS5B02G462100 | Chromosome 5B: 637,460,650-637,465,920 | - | exocytosis | exocyst |
| TGP | rs46500 | TGCAGGCATAGGCATGGCGCGGCCTCGCACAAGAGACGGAGTTGGTGGCAGCTTGGCCTGCTGG | TraesCS1B02G479300 | Chromosome 1B: 687,275,958-687,281,155 | protein kinase activity, protein binding, ATP binding | protein phosphorylation, regulation of meristem growth, floral organ development | integral component of membrane |
|  | rs10021 | TGCAGATTCATCGCTCGAGTTGTCATCTTCCGCCACTACCACCCTGCGTTGTCGGGCTGCTGCT | TraesCS2A02G034700 | Chromosome 2A: 15,185,525-15,223,170 | solute:proton antiporter activity | cation transport, transmembrane transport | integral component of membrane |
|  | rs3901 | TGCAGACCCTTGTACTCTGTCTGTGTTCACCCAAACACAACGGGGCATACCCTGGAACTCGCAC | TraesCS3B02G038800 | Chromosome 3B: 18,664,281-18,676,075 | oxidoreductase activity | oxidation-reduction process | - |
|  | rs5359 | TGCAGAGACGATTGGTCTGTTGGTTTCTCAGACTTGTGTGCACGTCGTCACCAGTATAAATACA | TraesCS4B02G364100 | Chromosome 4B: 652,414,628-652,416,298 | acid phosphatase activity | - | - |

Table S8. Continued.

| Trait | Marker | Sequence | Gene | Gene Position | Molecular function | Biological process | Cellular component |
| --- | --- | --- | --- | --- | --- | --- | --- |
| TGP | rs44371 | TGCAGGAGCGGCTGGCTTGCGGTGCGACCTTGGATGGCCTGGCCAGTGGCCAGGTCAGGTCAAT | TraesCS5D02G534000 | Chromosome 5D: 548,348,572-548,354,652 | protein kinase activity, ATP binding | protein phosphorylation | - |
|  | rs33214 | TGCAGCGTATGAGAAACGGTGGAATTACTATATACAAAGATATTTGGACGGGCACGTGTTAGCG | TraesCS7B02G191800 | Chromosome 7B: 329,795,959-329,796,165 | - |  | membrane, integral component of membrane |
| YLD | rs62854 | TGCAGTTCAAGTCCGTGACGCATAATGCCTATGTGGCTGCCGAGCAGGGTGGCGGCGGGGCCCT | TraesCS1D02G024100 | Chromosome 1D: 10,153,430-10,156,106 | hydrolase activity, hydrolyzing O-glycosyl compounds, actin filament binding, | carbohydrate metabolic process, actin filament organization, organic substance metabolic process | - |
|  | rs41511 | TGCAGCTTGGCAATCAGAACTACTTCATTTTTGAACTCCACTATACCTTGTTTCAGGGCCGTCT | TraesCS2A02G535100 | Chromosome 2A: 750,207,499-750,209,108 | protein kinase activity, ATP binding | protein phosphorylation | - |
|  | rs48834 | TGCAGGCTCATCAGGTGCTCGTAGGTGGATGGCCTGTCGCTGCCGAGATCGGAAGAGCGGGATC | TraesCS2B02G563200 | Chromosome 2B: 755,036,582-755,038,526 | iron ion binding, oxidoreductase activity, acting on paired donors, with incorporation or reduction of molecular oxygen, heme binding | oxidation-reduction process | - |
|  | rs474 | TGCAGAAACCTAGCTAGGGGCGGGTCGTGGCGGTGCCGAGATTACTGTAGCCGACCGAGATCGG | TraesCS3B02G454700 | Chromosome 3B: 696,332,309-696,333,657 | DNA-binding transcription factor activity, RNA polymerase II-specific, DNA-binding transcription factor activity, RNA polymerase II-specific, cis-regulatory region sequence-specific DNA binding, DNA binding, protein dimerization activity | transcription, DNA-templated, positive regulation of transcription by RNA polymerase II | nucleus |

Table S8. Continued.

| Trait | Marker | Sequence | Gene | Gene Position | Molecular function | Biological process | Cellular component |
| --- | --- | --- | --- | --- | --- | --- | --- |
| YLD | rs50412 | TGCAGGGCCTGAGGAGACGCATCCAGGGCTTGTTCACGCGGCTGCATGCCATGATCGCCGAGCG | TraesCS4A02G308600 | Chromosome 4A: 602,248,669-602,250,750 | monooxygenase activity, iron ion binding, oxidoreductase activity, acting on paired donors, with incorporation or reduction of molecular oxygen, heme binding | oxidation-reduction process | - |
|  | rs4017 | TGCAGACCGTGTACGCGTGCGGCGGCCGAAGGCATTAGGCCTGTGCGAGCTTTTCTCCTTTGTT | TraesCS4A02G485800 | Chromosome 4A: 739,272,852-739,276,534 | hydrolase activity, hydrolyzing O-glycosyl compounds | carbohydrate metabolic process | - |
|  | rs57710 | TGCAGTCAAGGTCGAACCCTCCGTGGCGTCCGCGAAGCTGCCGAGATCGGAAGAGCGGGATCAC | TraesCS5A02G428300 | Chromosome 5A: 613,374,133-613,374,591 | DNA binding, zinc ion binding | - | - |
|  | rs25292 | TGCAGCCGCCCTCTCGCCGTCGCTGCCGCTGCTGGTGGTGGTGCTACGAGGTCACTGCTCTATA | TraesCS6B02G359400 | Chromosome 6B: 631,193,221-631,194,157 | - | response to auxin | - |
|  | rs10538 | TGCAGATTTGTCGAGAGTGTGGAGTTGGACAAATGGAGGTAGCCAGGGACCATCTTAGCTAGTA | TraesCS6B02G451100 | Chromosome 6B: 710,001,532-710,007,317 | aspartic-type endopeptidase activity | - | integral component of membrane |
|  | rs52268 | TGCAGGTACATATCGAGCCCTCATCTCACCTCCATCTGCTTAGCCGTACTGCTCCTCGCAGGAA | TraesCS7A02G103000 | Chromosome 7A: 63,125,013-63,127,930 | protein kinase activity, calcium ion binding, ATP binding, polysaccharide binding | protein phosphorylation | integral component of membrane |
|  | rs15964 | TGCAGCAGAGCCGCTTGCTGCAACGCAGCCGATGCATGATGTGGTCGCTAGTGTTGCAGCACGC | TraesCS7B02G283400 | Chromosome 7B: 517,623,485-517,625,982 | transmembrane transporter activity | transmembrane transport | membrane, integral component of membrane |

Table S9. KEGG orthology-based annotation for regions surrounding GWAS significant

| Term | Database | ID | Input number | Background number | P-Value | Corrected P-Value | Input | Hyperlink |
| --- | --- | --- | --- | --- | --- | --- | --- | --- |
| Ribosome | KEGG PATHWAY | osa03010 | 2 | 358 | 0.07 | 0.35 | Os01g0896700|Os01g0896800 | http://www.genome.jp/kegg-bin/show_pathway?osa03010/osa:9271934%09red/osa:4325100%09red |
| Starch and sucrose metabolism | KEGG PATHWAY | osa00500 | 1 | 161 | 0.17 | 0.42 | Os08g0520900 | http://www.genome.jp/kegg-bin/show_pathway?osa00500/osa:4346068%09red |
| Plant hormone signal transduction | KEGG PATHWAY | osa04075 | 1 | 240 | 0.25 | 0.42 | Os02g0769100 | http://www.genome.jp/kegg-bin/show_pathway?osa04075/osa:4330855%09red |
| Biosynthesis of secondary metabolites | KEGG PATHWAY | osa01110 | 1 | 1177 | 0.76 | 0.94 | Os08g0520900 | http://www.genome.jp/kegg-bin/show_pathway?osa01110/osa:4346068%09red |
| Metabolic pathways | KEGG PATHWAY | osa01100 | 1 | 2290 | 0.94 | 0.94 | Os08g0520900 | http://www.genome.jp/kegg-bin/show_pathway?osa01100/osa:4346068%09red |
